# Progress in Wolff–Kishner reduction: developments toward milder and more efficient conditions

**DOI:** 10.1039/d6ra07179a

**Published:** 2026-11-04

**Authors:** Charan D. C., Nitinkumar S. Shetty

**Affiliations:** a Manipal Institute of Technology, Manipal Academy of Higher Education Manipal India nitin.shetty@manipal.edu

## Abstract

For over a century, the Wolff–Kishner reduction has been a cornerstone of organic synthesis, serving as a primary strategy for the selective deoxygenation of carbonyl groups into their corresponding methylene analogues. While classical protocols and their subsequent modifications are simple and broadly applicable, they are frequently limited by harsh reaction conditions, poor functional group tolerance, and substrate sensitivity, thus giving rise to alternative approaches of higher efficiency. This review provides a survey of the evolution of the Wolff–Kishner reduction from early modifications up to the most recent trends, which include metal catalysis, continuous flow techniques, microwave-induced methods, and solar-driven activation. In addition, mechanistic considerations regarding the decomposition of hydrazones and carbanion formation are also presented. Although significant steps towards the improvement of deoxygenation processes have been accomplished, further research is required due to the existing deficiencies in substrate compatibility, reagent demand, and pharmaceutical applications, outlining a distinct path forward for upcoming scientific investigations.

## Abstract 2

This review traces the Wolff–Kishner reduction from its classical conditions to modern, milder and more efficient variants, including metal-catalysed, microwave, flow and solar-driven methods, serving as a valuable reference for future research.

## Graphical abstract

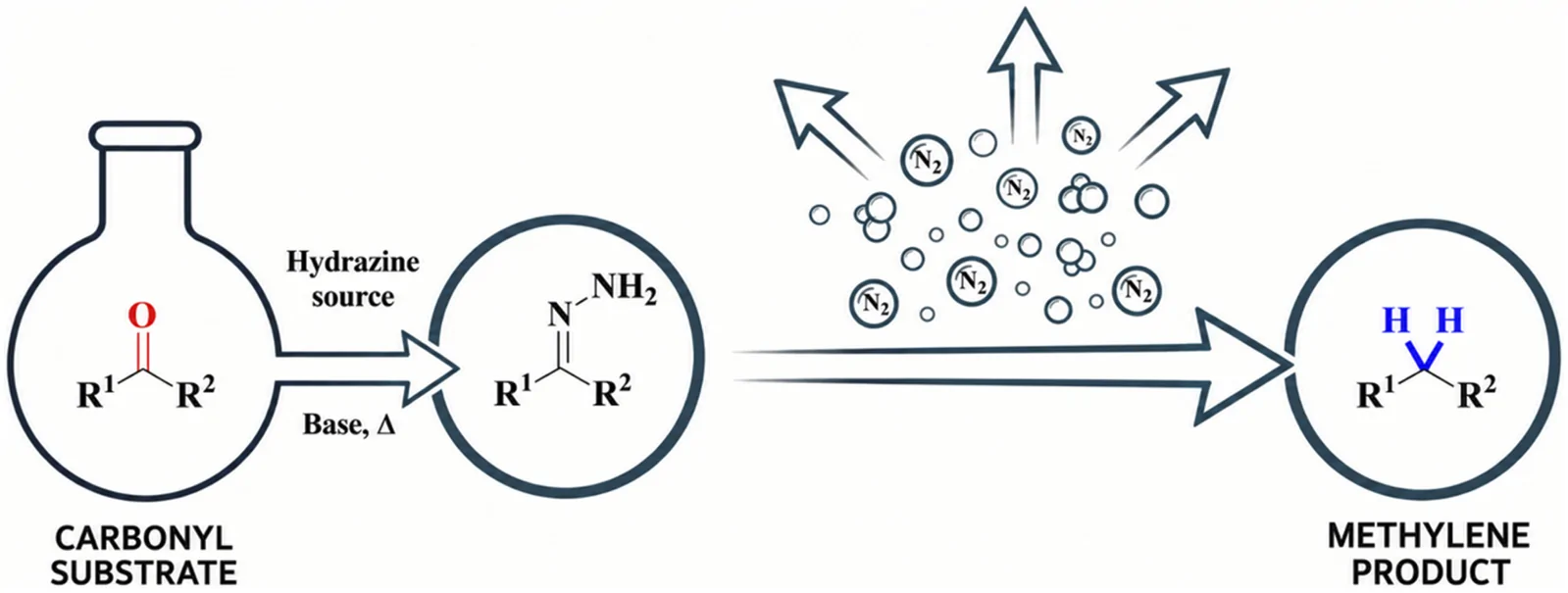

## 1. Introduction

Deoxygenation of carbonyl compounds stands as a cornerstone transformation in organic synthesis.^1^ Converting aldehydes and ketones into their corresponding methylene counterparts is an essential requirement encountered routinely during the synthesis of natural products, pharmaceuticals, and agrochemicals. In these contexts, carbonyl functionalities serve as pivotal synthetic intermediates yet must eventually be reduced to alkane frameworks in the final target structure.^2–4^ Meeting this recurring demand poses significant difficulties in designing synthetic routes towards structurally complex targets, highlighting the need for protocols capable of achieving deoxygenation with precision under rigorous conditions.^5,6^ Executing such a transformation while maintaining high chemoselectivity, accommodating diverse functional groups, and minimising by-product generation consequently remains a foremost objective in both academic research and industrial chemical synthesis.^7^

A number of approaches have been developed for the deoxygenation of carbonyls, each having unique mechanisms and operating principles. In the Clemmensen reduction, zinc amalgam acts as a reducing agent in strongly acidic media, which makes it complementary to basic systems but unsuitable for acid-sensitive substrates.^8^ Catalytic hydrogenation offers a metal-mediated alternative under neutral conditions, although this approach tends to suffer from excessive reduction and poor chemoselectivity in complex molecules.^9^ Hydride-based protocols allow milder conditions to be employed but suffer from reagent costs and limited applicability.^10–12^ Overall, no universal protocol has emerged, and the continuing need for reliable, economical deoxygenation procedures has fuelled research into the development of hydrazone-based approaches, most prominently the Wolff–Kishner reduction.^13^

The theory of carbonyl reduction reactions by means of intermediate hydrazones was proposed separately in 1911 by the Russian chemist Nikolai Kishner and in 1912 by the German chemist Ludwig Wolff; together, their work contributed to what is now known as the Wolff–Kishner reduction.^14,15^ Kishner showed that decomposition of preformed hydrazones prepared from aliphatic and aromatic ketones by base catalysis resulted in the corresponding hydrocarbon formation under highly alkaline conditions.^15^ Through his research with hydrazone and semicarbazone derivatives, Wolff further expanded this discovery, demonstrating that hydrazone intermediates can be deoxygenated at high temperatures and in strongly alkaline medium, resulting in products similar to those formed from Kishner's experiments.^14^ Although both approaches were highly attractive in theory, in practice they required sealed-tube apparatus, temperatures exceeding 180 °C, the use of stoichiometric amounts of hydrazine, and a strongly alkaline medium, thus placing severe restrictions on both techniques as they were not compatible with a great number of functional groups, particularly base-sensitive compounds such as esters and lactones due to their rapid hydrolysis,^16,17^ while sterically hindered carbonyl groups posed an additional challenge by impeding hydrazone formation entirely.^18^

The practical issues with the classical Wolff–Kishner protocol led to considerable refinements over the years. Early modifications by Huang-Minlon,^19^ Barton,^20^ Henbest,^21^ and Cram^22^ introduced variations in base identity, solvents, and reaction temperature. While these changes tackled certain shortcomings of the classical method, they retained the fundamental hydrazone-based mechanism. In spite of continuous improvement, all the approaches suffer from some significant shortcomings in terms of compatibility with various functional groups, substrate range, or practicality, necessitating the need for intrinsically milder deoxygenation methods. Addressing the liabilities of strongly alkaline media, Caglioti^23,24^ introduced a critical shift by achieving the hydride-driven reduction of tosylhydrazones near neutral conditions. Subsequent developments by Furrow and Myers^25^ improved functional group tolerance through reagent design, while Kuethe and co-workers^26^ showed the reaction could work at kilogram scale for a sterically demanding pharmaceutical intermediate, confirming its industrial relevance. Building on these findings, Wang and König^27^ demonstrated that visible-light-mediated radical addition to *N*-sulfonylhydrazones, followed by N_2_ loss, could generate α-functionalised carbanions for further derivatisation, thereby broadening the utility of the hydrazone platform well beyond conventional deoxygenation applications. These developments underscore that carbonyl deoxygenation, far from being a solved problem, continues to inspire innovation driven by the evolving demands of complex molecule synthesis.

Herein, we offer a comprehensive and critical survey of progress in Wolff–Kishner reduction chemistry over the decade spanning 2014 to 2025, covering both traditional modifications and cutting-edge synthetic contributions. Scheme 1 depicts the classical Wolff–Kishner reduction, along with its modifications spanning from Huang-Minlon to Myers. The central objective is to chart how this transformation has evolved from its operationally demanding roots towards gentler and more efficient protocols, highlighting how distinct and independently conceived strategies – including transition-metal catalysis with ruthenium, iridium, manganese and palladium (among them tandem dehydrogenation-Wolff–Kishner sequences that convert alcohols directly into hydrocarbons, and ruthenium-catalysed deoxygenative deuteration), continuous flow technology in silicon carbide reactors, solar-driven thermal activation, microwave-assisted processing, safer hydrazine surrogates such as methyl hydrazinocarboxylate, and metal-free or base-free protocols in aqueous or alcoholic media – have collectively broadened the applicability of this century-old reaction.

**Scheme 1:**
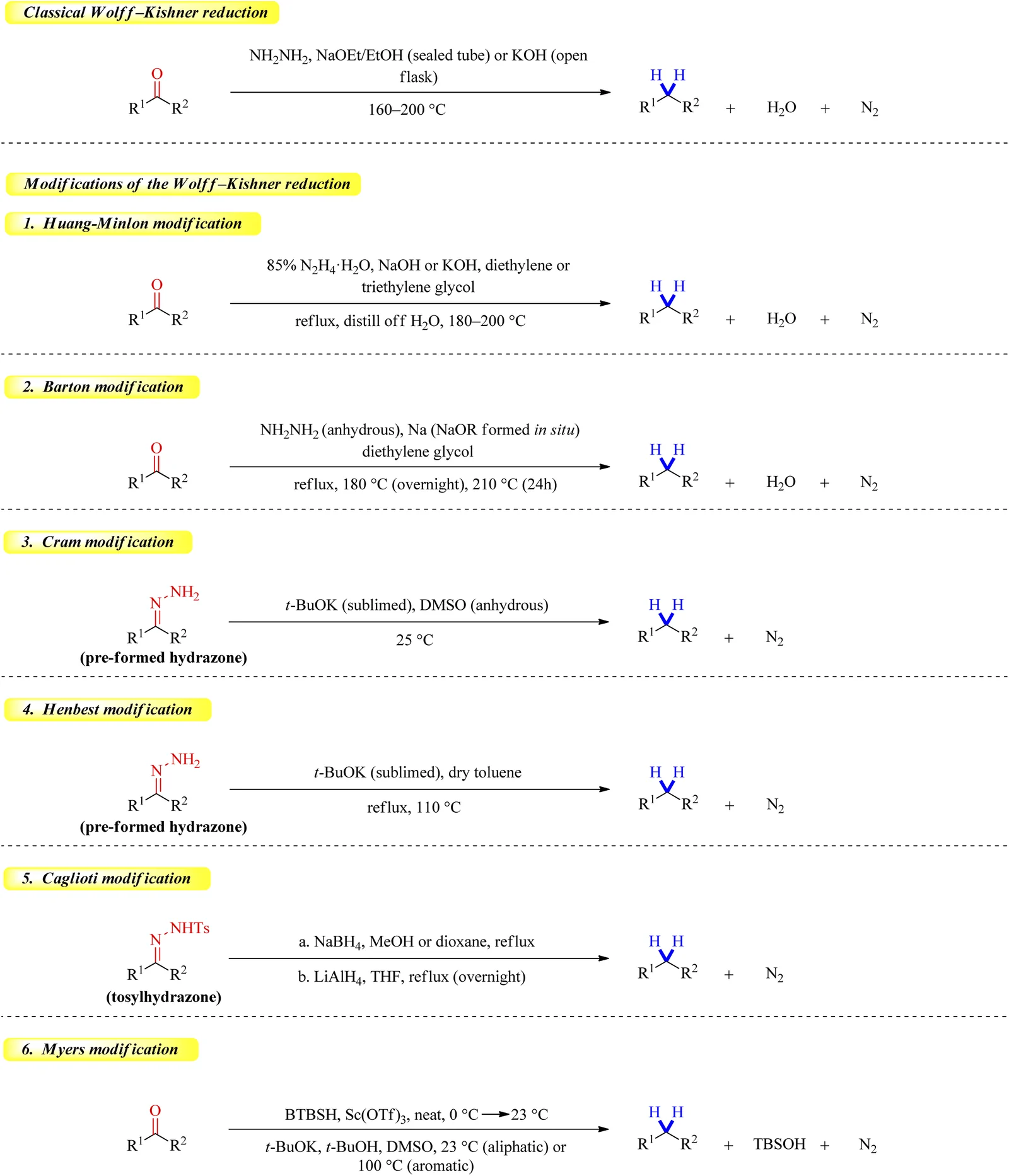
Classical Wolff–Kishner reduction and its modifications: Huang-Minlon, Barton, Cram, Henbest, Caglioti and Myers.

## 2. Modifications of the Wolff–Kishner reduction

### 2.1 Huang-Minlon modification

Huang-Minlon identified the water content of the reaction mixture, arising from hydrazone formation and from the hydrazine hydrate, as the principal factor responsible for reduced reaction temperatures and prolonged reaction times in existing glycol-based procedures. The modification introduced a distillation step between hydrazone formation and base-mediated decomposition, wherein the carbonyl substrate was refluxed in diethylene glycol with 85% hydrazine hydrate and base, followed by removal of aqueous distillate until the internal temperature reached 195–200 °C (typically 180–200 °C). Although metallic sodium was used in the initial trials, sodium hydroxide and potassium hydroxide proved equally effective and gave a simpler, more economical procedure, making them the bases of choice in practice. This eliminated the requirement for 100% hydrazine hydrate, reduced base loading substantially, and permitted large-scale operation with yields above 90% on a multi-hundred-gram scale. In a later report, Huang-Minlon extended the modified procedure to steroid ketones and observed that reduction proceeded normally at the C-3, C-7, C-12, C-17, and C-20 positions, whereas the C-11 keto group remained resistant to reduction under these conditions, as exemplified by 3-hydroxy-11-ketoetiocholanic acid. Moffett and Hunter subsequently established that this C-11 resistance could be overcome under forcing sealed-tube conditions, obtaining lithocholic acid in 82.4% yield *via* reaction of methyl 3(α)-acetoxy-11-ketocholanate with sodium methoxide and anhydrous hydrazine in methanol at 200 °C. Huang-Minlon also noted that base-sensitive substrates such as aldehydes and α,β-unsaturated ketones are best refluxed with hydrazine hydrate first, followed by slow addition of concentrated aqueous alkali, and an *o*-hydroxyaryl ketone gave an indazole instead of the methylene product.^19,28,29^

### 2.2 Barton modification

A more forcing version of the Wolff–Kishner reduction was developed by Barton and co-workers for substrates that do not respond to Huang-Minlon conditions. To keep moisture out of the reaction as far as possible, this method generates a strong base directly in the solution by reacting metallic sodium with diethylene glycol. Completely anhydrous hydrazine, prepared by refluxing hydrazine hydrate over sodium hydroxide pellets, is then distilled into this freshly formed sodium alkoxide medium, and the entire process is carried out in all-glass apparatus carefully protected from atmospheric moisture. The substrate is added to this medium and refluxed overnight at 180 °C, after which the temperature is raised to 210 °C by distilling some of the hydrazine back into the generator and the solution is refluxed for a further 24 h, enabling the reduction of hindered carbonyl groups. This technique was first used in the bulk synthesis of lanostanyl acetate from 7,11-dioxolanostanyl acetate in 69% yield and was further utilised in reducing 11-oxo steroids and hindered triterpenoid ketones, substrate types resistant to conventional Wolff–Kishner and Huang-Minlon reduction methods. Application of this forced procedure to a 19-oxoolean-12-ene, however, gave an abnormal outcome. Reduction of the model compound methyl 2α,3β-diacetoxy-19-oxoolean-12-en-28-oate under Barton's conditions afforded, after acetylation and methylation, not the expected olean-12-ene but a rearranged 13α*H*-olean-18-ene together with the α,β-unsaturated ketone 19-oxoolean-13(18)-ene, consistent with base-catalysed double-bond migration before deoxygenation.^20,30,31^

### 2.3 Cram modification

Efficient deoxygenation at room temperature was achieved through a notable Wolff–Kishner reduction variant introduced by Cram and co-workers, which utilises sublimed potassium *tert*-butoxide (*t*-BuOK) in anhydrous dimethyl sulfoxide (DMSO). To prevent the formation of competing azines and a subsequent drop in product yields, the pre-formed hydrazone was gradually introduced into the highly basic DMSO solution. Hydrocarbons are obtained in moderate to high yields (typically 60–90%), with immediate evolution of nitrogen as the hydrazone is added to the base, as illustrated by benzophenone hydrazone (90%), cyclohexanone hydrazone (80%), benzaldehyde hydrazone (67%) and camphor hydrazone (64%). The superior reactivity observed in DMSO is attributed to the much higher reactivity of anions in this solvent than in hydroxylic solvents of comparable dielectric constant, which enhances the activity of the alkoxide toward the N–H bond and promotes the base-catalysed isomerisation of the hydrazone, proposed to be the rate-limiting step, followed by loss of molecular nitrogen. Despite these advantages, the requirement for pre-formed hydrazones and slow addition (8 h for 2 g of benzophenone hydrazone) detracts from the operational simplicity of the method.^22^

### 2.4 Henbest modification

The innovation adopted by Henbest and colleagues involved replacing the conventional conditions (metal alkoxides at 180–220 °C or KOH in ethylene glycol at about 200 °C) with sublimed *t*-BuOK in dry toluene. Using a powerful base in an anhydrous environment enables the reaction to take place at a markedly lower temperature (benzophenone hydrazone gave diphenylmethane in 85% yield after 4 h in refluxing toluene, about 110 °C), which may avoid the anomalous reactions attributed to the vigorous classical conditions. Two substrates that give abnormal products under conventional conditions were reduced normally. Cholest-4-en-3-one hydrazone gave cholest-4-ene in 65% yield (81% from the semicarbazone) with less than 10% of cholest-3-ene, and an α-aminoketone hydrazone gave the normal amine in 83% yield with only a trace of the elimination product. Contrary to the Cram procedure, no slow or controlled addition of hydrazone is needed, thus simplifying experimental handling and improving practicality. The authors regarded the amino ketone result as a striking example of the effect of an anhydrous medium, which could not be explained by the lower temperature alone, and suggested that the procedure may be applicable to other α,β-unsaturated ketones.^21^

### 2.5 Caglioti modification

Modification of the Wolff–Kishner reduction procedure by Caglioti allows for the reduction of tosylhydrazones into alkanes through hydride-transfer agents like lithium aluminium hydride and sodium borohydride under mild conditions, without strong acids or bases. Alicyclic and steroidal ketones and aliphatic aldehydes are reduced by NaBH_4_ in good yields (up to 80%), whereas aromatic ketones such as α-tetralone give only low yields (30–35%). With NaBH_4_, methanol and dioxane gave comparable yields (55–80% for aliphatic and steroidal substrates), although the 17- and 20-keto steroid tosylhydrazones gave the saturated hydrocarbon (60–70%) only in dioxane. LiAlH_4_, in contrast, reduces steroidal ketones to the corresponding alkanes in moderate yields (40–18070%) and also converts aromatic aldehydes into the corresponding methyl compounds. Mechanistic studies indicate that reduction of the tosylhydrazone with NaBH_4_ affords an isolable alkyl-tosylhydrazide intermediate (isolated in 60–70% yield from cyclohexanone tosylhydrazone), which subsequently undergoes elimination of *p*-toluenesulfinate to generate an unstable alkyl diimide that decomposes to nitrogen and the corresponding alkane. In aprotic solvents, the same intermediate was obtained with NaBH_4_ (up to 75% in diglyme) and with diborane (55–60%). Beyond ketones and aldehydes, the method was extended to acyl-tosylhydrazides derived from carboxylic acids, providing a route for the direct reduction of a carboxyl group to a methyl group, as demonstrated for stearic acid and 3α-acetoxycholanic acid, although aromatic acyl-tosylhydrazides gave comparatively low yields. A notable limitation of LiAlH_4_ is that the tosylhydrazones of 17-keto steroids gave Δ^16^-olefins (60–70%) rather than the saturated hydrocarbon, analogous to the Bamford–Stevens reaction, and tosylhydrazones of α,β-unsaturated steroidal ketones gave olefin mixtures with shifted double bonds (Table 1).^32–35^

**Table 1:** Evolution of Wolff–Kishner reduction modifications: a comparative timeline

	Huang-Minlon	Barton	Cram	Henbest	Caglioti	Myers
Year introduced	1946	1955	1962	1963	1964	2004
Hydrazine source	85% Hydrazine hydrate	Completely anhydrous hydrazine	Pre-formed hydrazone	Pre-formed hydrazone or semicarbazone	Tosylhydrazone (isolated, or formed *in situ* from TsNHNH_2_)	1,2-Bis(*tert*-butyldimethylsilyl)hydrazine
Base/reductant	NaOH or KOH	Na metal → NaOR (*in situ*)	*t*-BuOK (sublimed)	*t*-BuOK (sublimed)	LiAlH_4_; NaBH_4_	Sc(OTf)_3_ (cat., condensation); *t*-BuOK/*t*-BuOH (reduction)
Solvent	Diethylene glycol or triethylene glycol	Diethylene glycol (redistilled)	DMSO (anhydrous)	Dry toluene	THF (LiAlH_4_); MeOH or dioxane (NaBH_4_)	Neat (condensation); DMSO (reduction)
Temperature	180–200 °C	180 °C → 210 °C (two-stage)	25 °C	110 °C	Room temp. to reflux (*e.g.* ∼66 °C in THF)	0 °C → 23 °C (condensation); 23–100 °C (reduction)
Substrate scope	Ketones, aldehydes, α,β-unsaturated carbonyls, keto acids and steroid ketones (C-3, C-7, C-12, C-17, C-20)	Sterically hindered ketones (*e.g.* 11-oxo-steroids, hindered triterpenoid ketones)	Aldehydes and ketones as pre-formed hydrazones	Benzophenone and ketones that give abnormal products under conventional conditions	Alicyclic and steroidal ketones, aliphatic and aromatic aldehydes; acyl-tosylhydrazides (RCO_2_H → RCH_3_)	Broad; aliphatic and aromatic aldehydes and ketones, tolerating ketals and amines
Key advantage	Large-scale; cheaper 85% hydrazine hydrate suffices	Reduces hindered carbonyls that resist Huang-Minlon conditions	Room temperature operation	Milder than classical conditions; normal reduction of substrates that give abnormal products under classical conditions	Mild, no strong acid or base; one-pot from the carbonyl possible	Avoids isolation of labile hydrazones; mild conditions
Key limitation	Does not reduce the C-11 steroid ketone; base-sensitive substrates are best treated with slow alkali addition	Forcing conditions (overnight at 180 °C, then 24 h at 210 °C), rigorous exclusion of water, and a 19-oxoolean-12-ene gave a rearranged product	Slow addition required; azine by-product	Requires preformed hydrazone (*in situ* formation gives azine); long reaction times of 4–60 h	LiAlH_4_ – 17-keto steroids give Δ^16^ olefins, and enones give isomer mixtures; NaBH_4_ – low yields for aromatic carbonyls	Reagent must be prepared from anhydrous hydrazine; slow at 23 °C for aromatic or hindered substrates; Kishner–Leonard elimination with α-heteroatom substrates; silyl ether cleavage under basic conditions

### 2.6 Myers modification

A considerable improvement of the Wolff–Kishner reduction was accomplished by Furrow and Myers, who substituted hydrazine with 1,2-bis(*tert*-butyldimethylsilyl)hydrazine (BTBSH), a stable alternative. Using a catalytic Lewis acid, typically scandium triflate, BTBSH condenses with carbonyl compounds to form *N-tert*-butyldimethylsilylhydrazone (TBSH) intermediates, which are known to be considerably more stable and less prone to unwanted azine formation than conventional hydrazones. Subsequent reaction with *t*-BuOK in the presence of *t*-BuOH (as a proton source) in DMSO converts these TBSH intermediates into the corresponding hydrocarbon, with loss of nitrogen gas and *tert*-butyldimethylsilanol (TBSOH), the by-product of the earlier condensation. A distinguishing feature of this protocol is that the entire sequence can be conducted in a single reaction flask without isolating or purifying the TBSH intermediate, with reduction proceeding at markedly lower temperatures (23–100 °C) than traditional Wolff–Kishner conditions. Most aliphatic substrates, such as 4-(4-methoxyphenyl)-2-butanone (93%), are reduced efficiently at 23 °C, whereas aromatic substrates such as 3-phenoxybenzaldehyde (96%) and 6,7-dimethoxy-1-tetralone (92%) require 100 °C. Overall, this modification enables efficient deoxygenation under significantly milder and operationally convenient conditions compared to earlier Wolff–Kishner variants, although substrates with α-heteroatom substituents can undergo competing Kishner–Leonard elimination and base-labile groups such as silyl ethers may be cleaved.^25^

## 3. Mechanistic insights into the Wolff–Kishner reduction

The mechanistic picture of the Wolff–Kishner reduction was established through systematic kinetic investigations by Szmant and co-workers spanning 1952 to 1977. Early kinetic studies on diaryl ketone hydrazones in butyl carbitol demonstrated strict second-order kinetics, first order each in hydrazone and base, confirming that rapid reversible deprotonation of the terminal N–H by alkoxide generates the hydrazone anion as the obligatory first step. The rate-determining step was assigned to the subsequent concerted process within this anion, involving proton capture at the carbon terminal coupled with solvent-assisted N–H abstraction at the nitrogen terminal, generating a diimide-type species that rapidly expels N_2_ to release a carbanion, which is then reprotonated by solvent to furnish the alkane.^18,36^ The wide range of enthalpies (15.7 to 37.4 kcal mol^−1^) and entropies (−31.0 to +13.8 eu) of activation indicated that this hydrogen shift can proceed *via* either an intramolecular cyclic pathway or an intermolecular solvent-mediated relay, depending on substrate geometry. Hammett analysis across aryl hydrazones revealed a nonlinear concave-downward plot, with *ρ* ≈ +1.0 for mildly electron-withdrawing groups and *ρ* ≈ −0.8 for strongly electron-withdrawing groups, reflecting competing electronic demands at both termini of the hydrazone anion in the transition state (Scheme 2).^18^

**Scheme 2:**
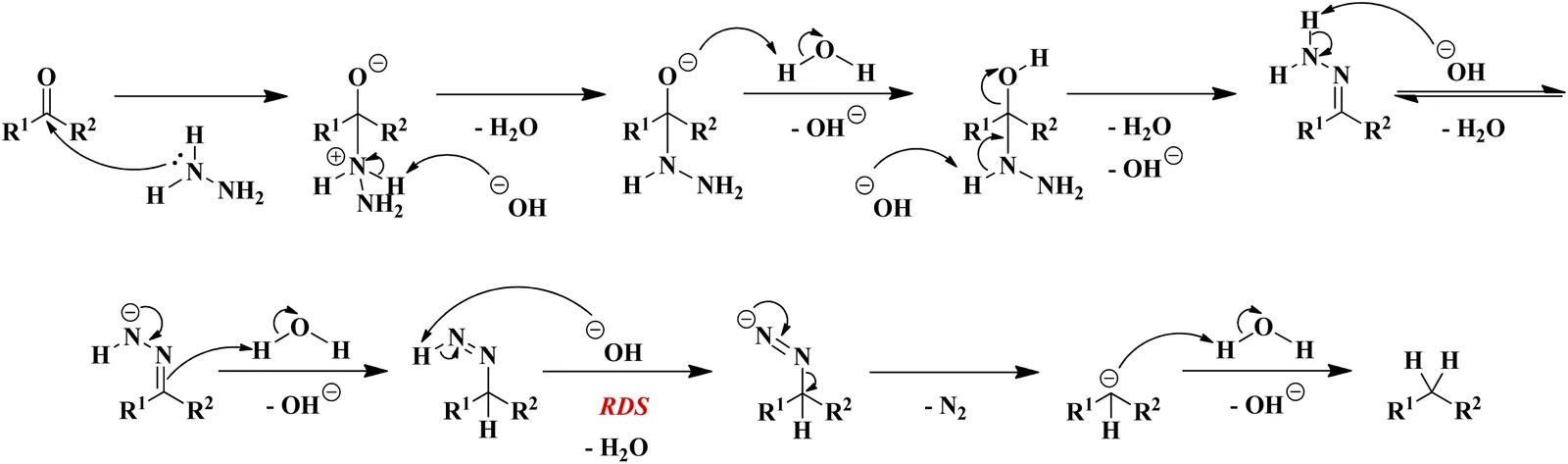
Proposed mechanism of the Wolff–Kishner reduction under classical basic conditions.

The pronounced dependence of the Wolff–Kishner rate on solvent composition was demonstrated by studying the reaction in mixtures of butyl carbitol and DMSO across the temperature range 100 to 195 °C. Addition of DMSO accelerated the reaction by lowering the enthalpy of activation, but the rate passed through a sharp maximum before declining steeply as the protic component was depleted, and in absolute DMSO the rate constant fell approximately 70-fold relative to systems containing only a residual trace of hydroxylic solvent (∼0.12 M). This confirmed that at least one molecule of protic solvent participates directly in the rate-determining transition state, functioning as a proton source at the carbon terminal while DMSO acts as a superior hydrogen-bond acceptor at the nitrogen terminal. Stereochemical considerations established that the *trans* hydrazone anion is the kinetically preferred intermediate in protic media, with *ortho* substituents capable of hydrogen bonding stabilising the *cis* anion instead and leading to pronounced rate acceleration. Comparative kinetic studies on benzophenone, fluorenone, dibenzotropone, and dibenzosuberone hydrazones further showed that rates are governed primarily by enthalpy differences, with fluorenone hydrazone reacting approximately 94-fold faster than benzophenone hydrazone at 150.5 °C owing to exceptional stabilisation of the fluorenyl carbanion at the carbon terminal.^17,37,38^

The mechanistic question of whether N_2_ extrusion generates a carbanion or a free radical intermediate was resolved experimentally by Taber and Stachel through an elegant substrate-probe strategy. Three structurally equivalent δ,ε-unsaturated precursors were prepared: a bromide for radical cyclisation, an iodide for anionic cyclisation, and a ketone for Wolff–Kishner reduction. Radical conditions gave a 2.2 : 1 *cis*-selective cyclisation ratio, whereas anionic conditions gave a 1 : 10.3 *trans*-selective ratio. The Wolff–Kishner product ratio of 1 : 11 (among the ∼5% of material that underwent cyclisation) matched the anionic pathway almost exactly, providing the first direct experimental evidence for an sp^3^-hybridised carbanion intermediate under classical basic conditions. Critically, reduction of the corresponding tosylhydrazone under mild NaBH_4_/acetic acid conditions gave a 3 : 1 *cis*-selective ratio, demonstrating that the carbanion *versus* radical pathway is condition-dependent rather than mechanistically universal.^39^

Computational investigation by Yamabe and co-workers^40^ provided the most mechanistically complete picture of the reduction to date. DFT calculations established that hydrazone formation proceeds without base assistance, requiring only modest activation energy through solvent-mediated proton transfer circuits. However, N_2_ extrusion without base is computationally impossible, with activation barriers exceeding 42 kcal mol^−1^ – unambiguously confirming the obligatory role of alkoxide catalysis at this stage. Most significantly, the calculations revealed a substrate-dependent mechanistic bifurcation at the diimine intermediate. For acetone, N_2_ loss and C–H bond formation are concerted through an E2-type transition state, producing propane directly without a discrete carbanion. For acetophenone, phenyl-ring stabilisation of the developing negative charge permits formation of a genuine carbanion intermediate, which undergoes protonation at the *para* position followed by a [1,5]-hydrogen shift to yield ethylbenzene. Taken together, the Wolff–Kishner reduction does not follow a single universal mechanism, with the operative pathway instead depending on how well the substrate can stabilise anionic charge at the carbon centre after loss of dinitrogen.

## 4. Recent advances and contemporary approaches in the Wolff–Kishner reduction

Newman and co-workers^41^ reported the development of a silicon carbide (SiC) microreactor specifically engineered to address limitations in applying flow chemistry to fine chemical synthesis. Ceramic microreactors exist, but their use remains limited. This design addresses that limitation by combining chemical inertness, corrosion resistance, and gas-tight sealing to make the system practical under demanding conditions. As a demonstration, the reduction of benzophenone to diphenylmethane was used to validate reactor performance, with operation in a pressurised, headspace-free environment enabling efficient conversion with significantly reduced hydrazine loading.

Two key advantages emerge from these conditions – reduced hydrazine usage, which minimises cost, hazards, and waste; and shortened reaction times by two orders of magnitude, reflecting the intensified conditions achievable under continuous flow. The suppression of hydrazine loss under pressure maintains reactive equilibria, thereby limiting side reactions such as azine formation – an issue observed under lower pressure conditions and in conventional batch setups. Substrate scope assessment demonstrates that the approach is effective across a range of aldehydes and ketones, while the absence of any signs of corrosion during prolonged usage is indicative of the robustness and scalability of the reactor. In summary, this evidence portrays the SiC microreactor as a promising intermediate solution between laboratory synthetic experiments and industrial applications, providing an environmentally friendly alternative to conventional reduction strategies that use hydrides, silanes, or metals (Scheme 3).

**Scheme 3:**
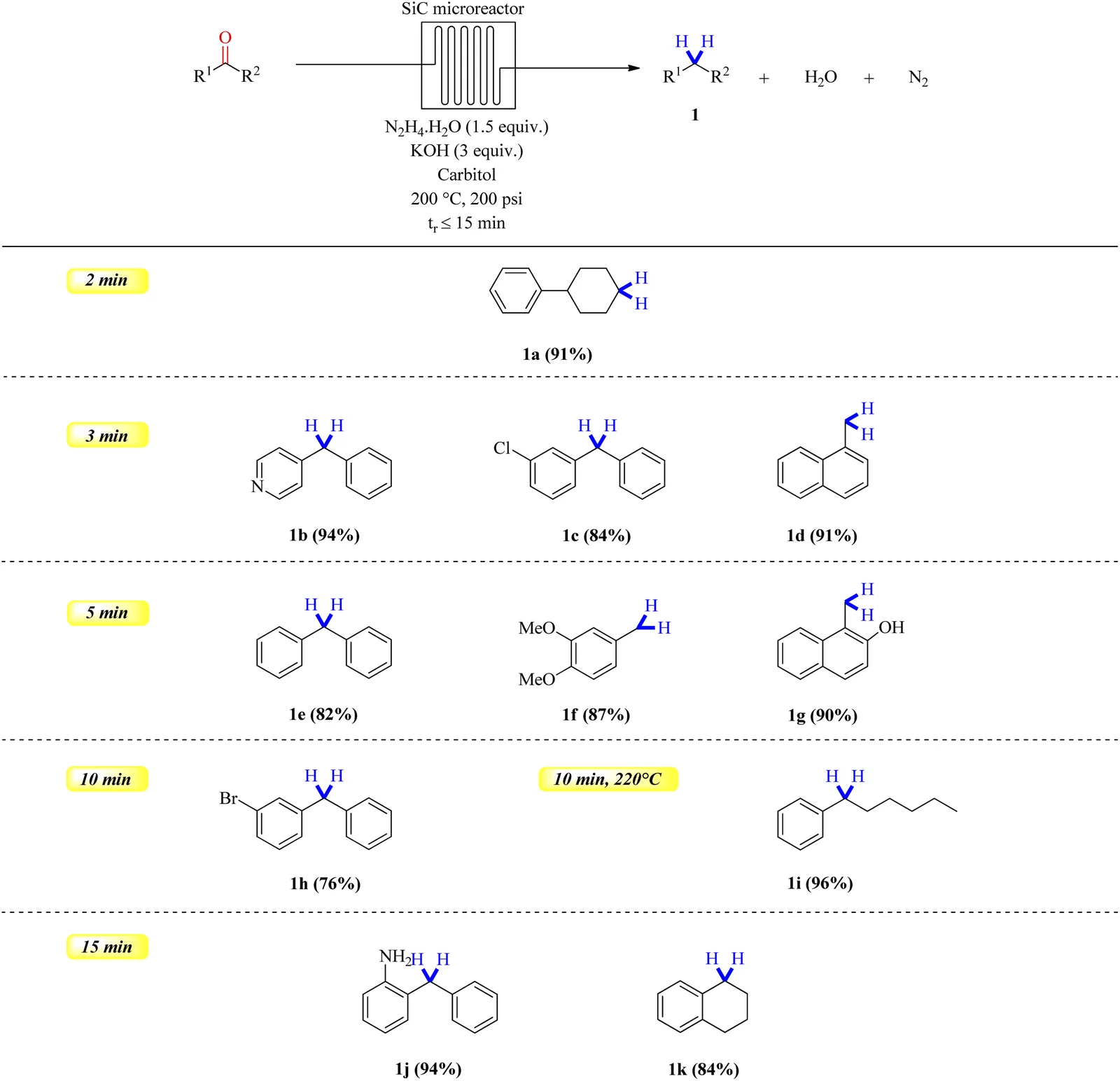
Substrate scope of Wolff–Kishner reductions performed in a silicon carbide microreactor.

The goal of Agee *et al.*^42^ was to determine whether a solar reflector could replace the conventional electrical heater as a viable thermal source for conducting the Wolff–Kishner reaction. The solar reflector setup was based on a previously developed system,^43^ in which a repurposed satellite dish coated with reflective Mylar tape had been used as a solar heat source to drive a Friedel–Crafts acylation, reaching temperatures in excess of 300 °C. However, the aluminium heating block was removed to avoid heat loss and thereby improve thermal efficiency. Furthermore, the round-bottom flask was painted black to ensure maximum absorption of solar energy, while minimising any interference from photochemical side reactions. High-boiling solvents were used in the experiment to show that the reflector could produce temperatures as high as 235 °C, indicating that there was enough heat energy to drive the Wolff–Kishner reductions to completion. These findings demonstrate that the system is not merely conceptual but offers a viable, practical alternative to conventional heating methods.

Comparative analysis showed that the new technique gave yields similar to those achieved using traditional heating methods, indicating that the sustainability gains did not come at the expense of process efficacy. Further mechanistic analyses revealed that the use of hydrazine hydrate in place of hydrazine produced identical results, indicating that safer reagent forms can be utilised without a loss in efficiency. Energy consumption analysis showed that the classical Wolff–Kishner reduction required about 1.20 kWh, comprising 1.07 kWh for the reflux step and 0.13 kWh for purification. The solar-assisted method eliminated the reflux-step energy requirement entirely, so only the 0.13 kWh purification cost remained. These data suggest significant energy savings, rendering the process a more sustainable alternative (Scheme 4).

**Scheme 4:**
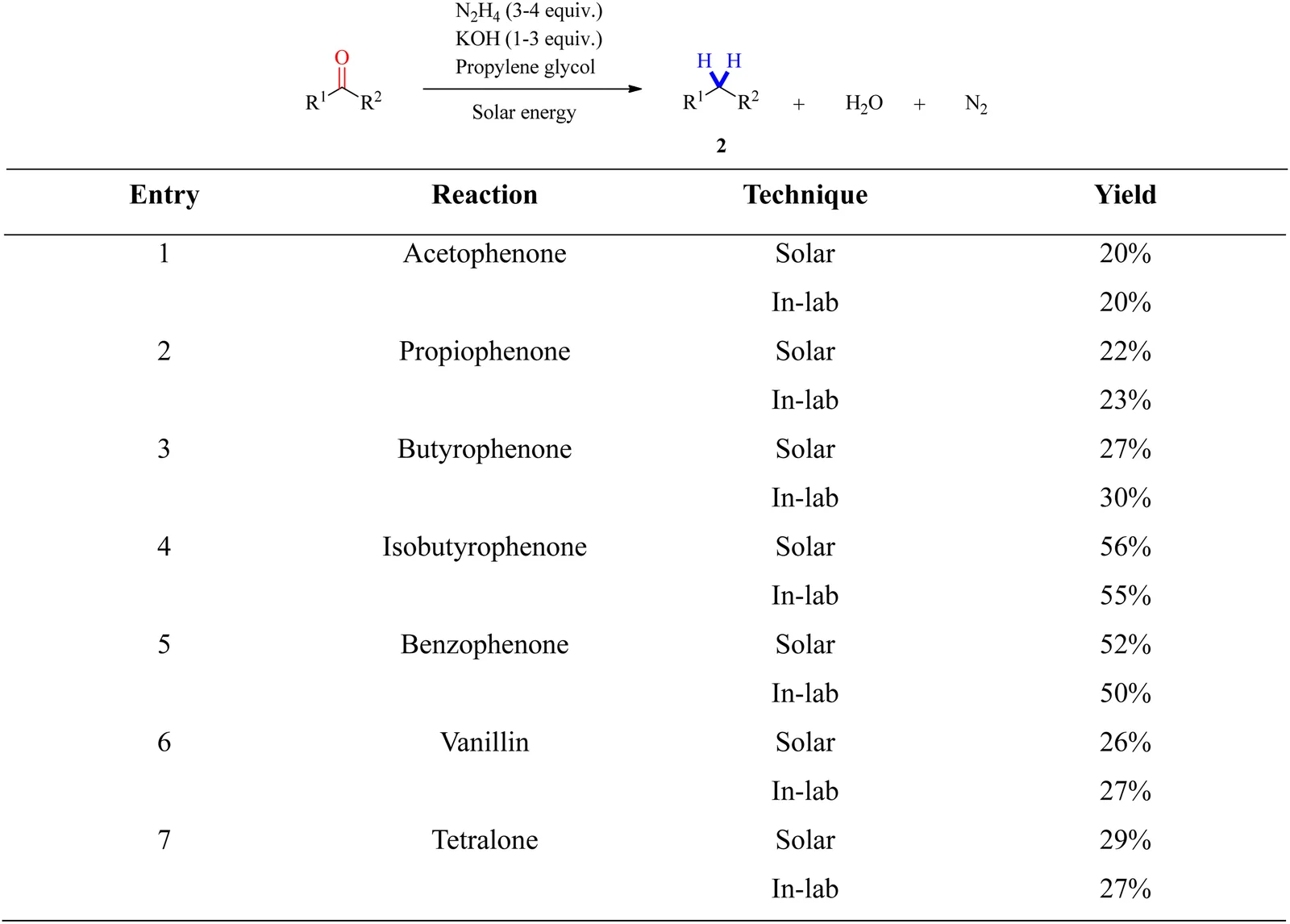
Evaluation of solar-irradiated *versus* electrically heated Wolff–Kishner reductions of carbonyl compounds.

A procedure for the formation of methylene compounds from aromatic aldehydes and ketones *via* a readily isolable carbomethoxyhydrazone intermediate has been proposed by P. B. Cranwell *et al.*,^44^ minimising the use of hydrazine in excess amounts. In this process, the carbonyl compound undergoes initial condensation with methyl hydrazinocarboxylate in ethanol with acetic acid as the catalyst, leading to the formation of bench-stable intermediates that can be easily isolated. To ensure rapid and efficient decomposition of these hydrazones, the KOH required predissolution in the solvent prior to addition of the hydrazone. The method works for substituted acetophenones, diaryl ketones, and aromatic aldehydes, though steric congestion lowers yield and promotes competitive azine formation. At elevated temperatures, electrophilic intermediates form, followed by hydroxide addition and irreversible decarboxylation to give the active amide species, consistent with the effervescence observed. Nitrogen extrusion then follows more slowly and defines the rate-limiting step of the transformation (Scheme 5).

**Scheme 5:**
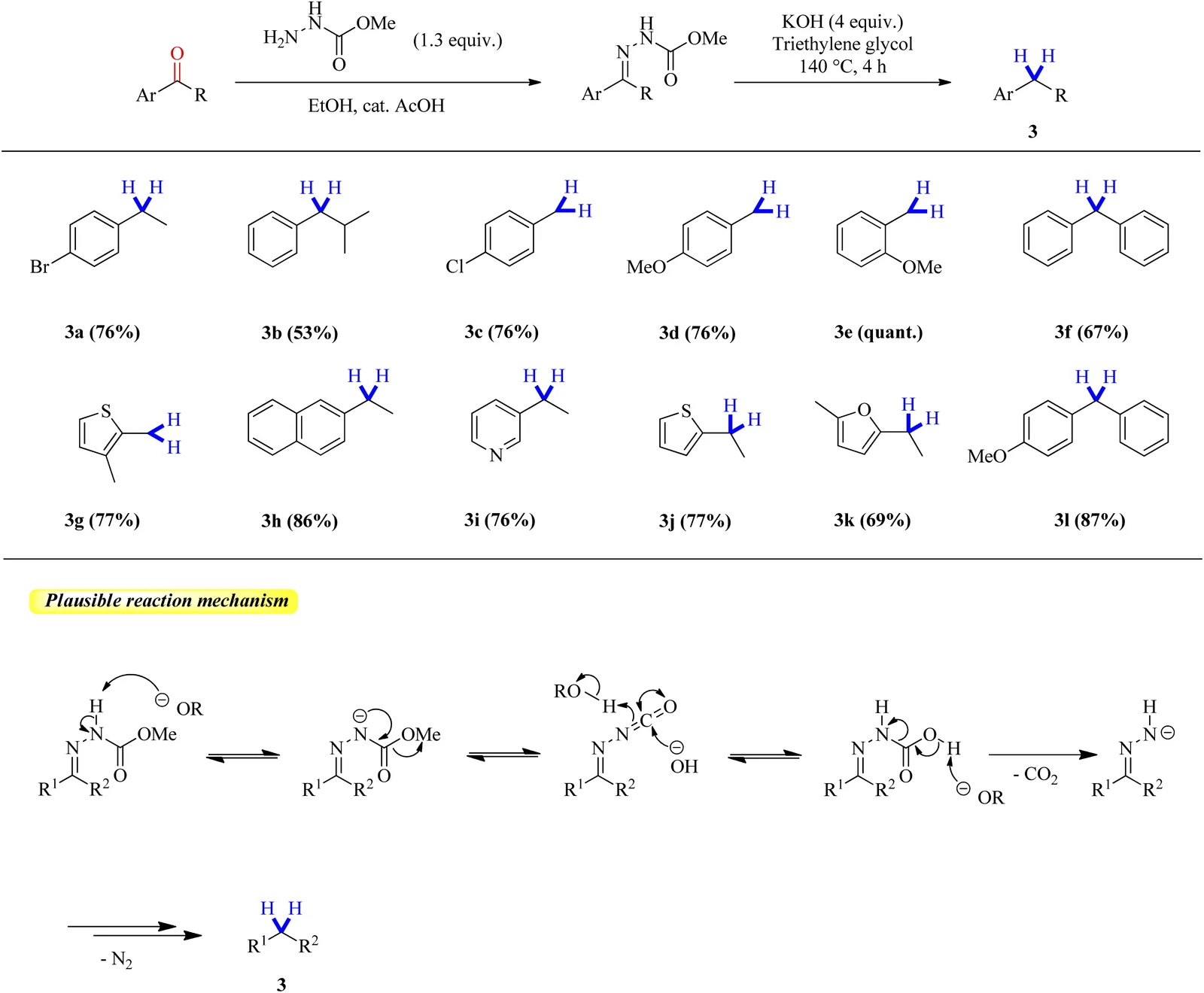
Wolff–Kishner reduction utilising methyl hydrazinocarboxylate as a safe reagent alternative.

The following year, Cranwell and Russell^45^ adapted their carbomethoxyhydrazone-based protocol for use in an undergraduate laboratory setting, where the conversion of acetophenone to ethylbenzene was carried out by several groups of students to assess feasibility and reproducibility. The reduction was carried out under similar basic conditions, and the product was purified *via* a silica plug. The carbomethoxyhydrazone intermediate, synthesised as earlier outlined, was analysed using IR spectroscopy, melting point analysis, and ^1^H NMR (with and without D_2_O shake), while the reduced product was characterised by ^1^H NMR and ^13^C NMR (including a DEPT135 experiment for carbon-type assignment). Among a class of forty students, consistent yields for the intermediate compound were obtained, whereas the reduction provided moderate to good yields. Notably, the average yield of ethylbenzene (57%) was reasonably consistent with the instructor's yield (70%), thereby validating the efficiency and reliability of the procedure (Scheme 6).

**Scheme 6:**
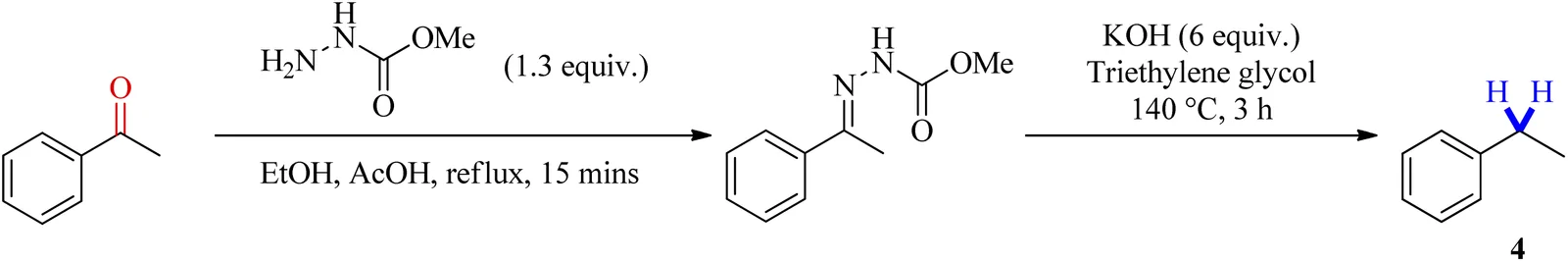
Reduction of acetophenone to ethylbenzene using methyl hydrazinocarboxylate as a hydrazine surrogate.

A catalytic redox strategy that integrates dehydrogenation with Wolff–Kishner reduction was conceived by Dai *et al.*,^46^ thereby enabling direct C(sp^3^)–O defunctionalisation with improved step economy. Initial experiments conducted on piperonyl alcohol using Vaska's complex (*trans*-[IrCl(CO)(PPh_3_)_2_]) proved the viability of the process, though a highly concentrated reaction solution was later found necessary to drive the reaction to completion, which would ultimately limit practical scalability. However, the significantly reduced reactivity towards aliphatic compounds, as well as elevated temperature and pressure requirements under closed-system conditions, highlights certain limitations regarding the range of potential substrates and the applicability of such an approach. Hence, ruthenium catalysis was considered a preferable option. The dinuclear complex [Ru(*p*-cymene)Cl_2_]_2_ with bidentate phosphines was found to be superior to Vaska's complex and other pincer complexes, with careful control of DMSO loading proving critical, as excess DMSO coordinated to and saturated the ruthenium centre, suppressing turnover until its use was reduced from bulk solvent to a catalytic additive.

Further optimisations revealed that 1,2-bis(dimethylphosphino)ethane (dmpe) was crucial for enhancing reactivity, highlighting the importance of bite angle in the ligand. The use of *t*-BuOH as solvent alongside a catalytic quantity of DMSO was found to be important, with DMSO engaging in hydrogen-bonding interactions with *t*-BuOH that supported the productive coordination geometry at ruthenium. Mechanistically, Ru-catalysed dehydrogenation of the system produces an aldehyde, which forms a hydrazone that is subsequently decomposed by base to produce hydrocarbons, along with the evolution of H_2_ and N_2_. Unlike classical variants, this method shows significant chemo- and regioselectivity, utilising catalytic DMSO and a substoichiometric base to preferentially deoxygenate the least sterically hindered alcohol-derived C–O bond in multifunctional substrates (Scheme 7).

**Scheme 7:**
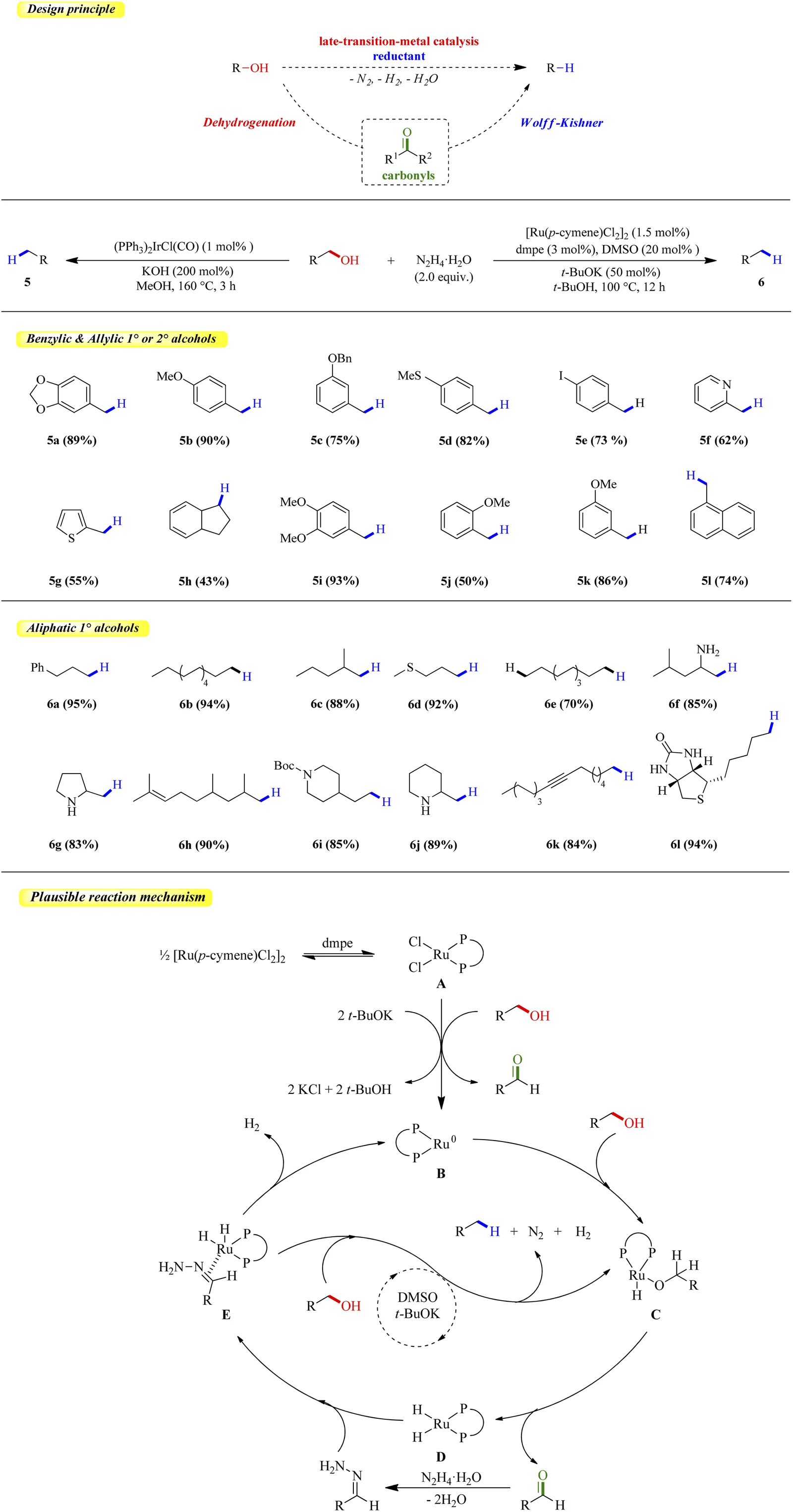
Iridium- and ruthenium-catalysed direct deoxygenation of benzylic, allylic, and aliphatic alcohols.

To access methylene derivatives, a strategy involving the photo- and Co(i)-catalysed [2 + 2 + 2] cycloaddition of diynes with nitriles was first employed by Fischer *et al.*^47^ to generate a heterobiaryl ketone intermediate, which was then deoxygenated to yield the corresponding methylene-containing heterobiaryl. Early attempts to achieve reduction using a tosylation-LiAlH_4_ sequence or base-mediated Wolff–Kishner conditions were unsuccessful. The tosylation-LiAlH_4_ sequence did not afford the desired product, while the subsequent attempt using base-mediated Wolff–Kishner conditions proceeded *via* the hydrazone intermediate but suffered from significant decomposition to undesired side-products under aqueous KOH. To overcome this, a modified version of the Wolff–Kishner procedure was used, where hydrazine hydrate in diglyme at 180 °C was subjected to microwave radiation. Such conditions enabled the effective formation of the hydrazone, which was then further decomposed in the absence of any base, affording the methylene product in 83% yield. The reaction achieved deoxygenation by first forming a hydrazone and subsequently releasing N_2_ gas. By completely removing the base from the system, unwanted side-products were effectively suppressed. Additionally, the resulting product slowly oxidises back into the starting ketone, indicating that the system is inherently redox-reversible under ambient conditions (Scheme 8).

**Scheme 8:**
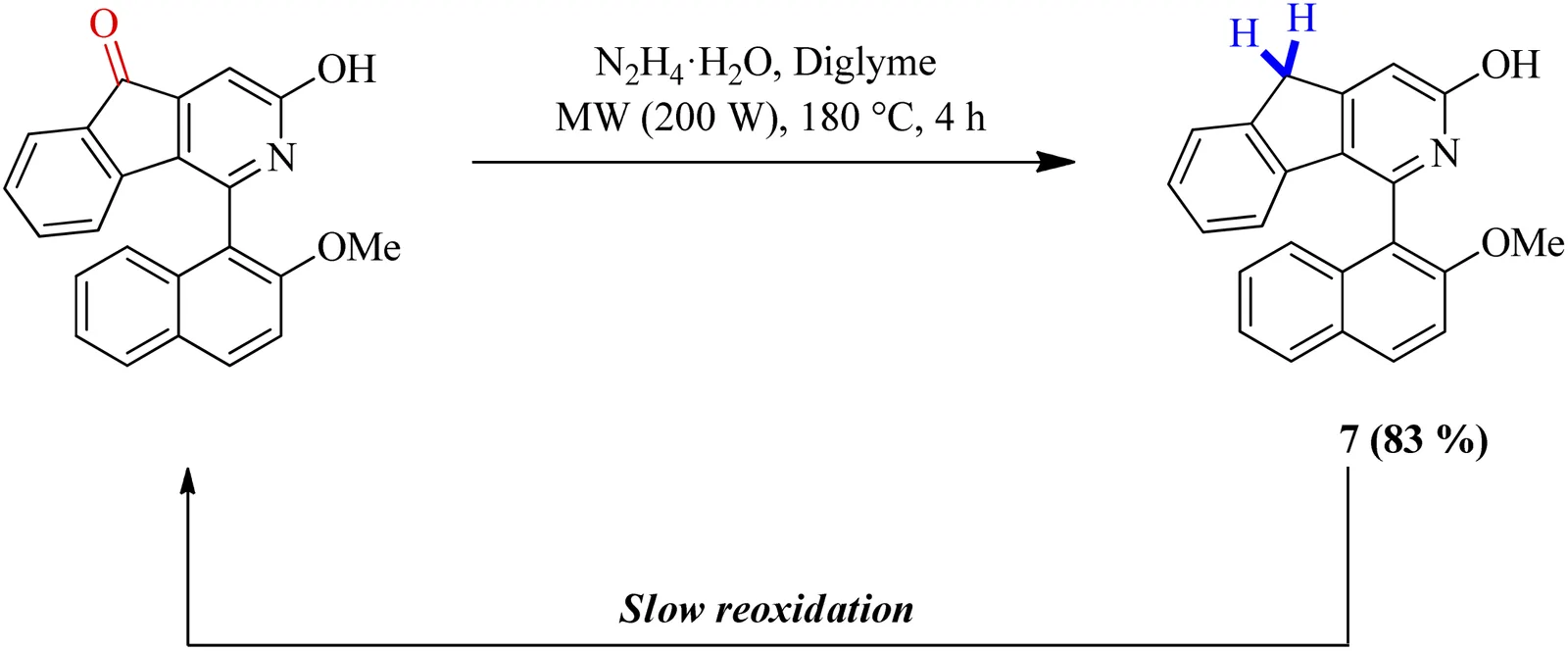
Microwave-assisted, base-free Wolff–Kishner reduction of a heterobiaryl ketone intermediate.

A manganese complex bearing a PNP-pincer ligand was presented by Bauer *et al.*^48^ to catalyse the direct deoxygenation of benzylic and aliphatic primary alcohols *via* a tandem oxidative dehydrogenation/Wolff–Kishner process leading exclusively to H_2_, N_2_, and H_2_O by-products. Catalyst preparation involved the use of *i*Pr-PNHP ligand in combination with Mn(CO)_5_Br to afford the Mn(*i*Pr-PNHP)(CO)_2_Br complex, which was further subjected to reduction with NaHBEt_3_, yielding the Mn(*i*Pr-PNHP)(H)(CO)_2_ catalyst. Efficient conversion is achieved in *t*-BuOH media and *t*-BuOK conditions, with the closed-system having no effect on the activity of the reaction, suggesting easy H_2_ evolution during the process. A wide variety of primary benzylic alcohols containing different substituents, both electron-donating and electron-withdrawing groups, together with primary aliphatic alcohols serve as substrates for the reaction, giving good to high yields (typically 83–99%), while secondary alcohols remain largely unreactive. The proposed mechanism of the reaction involves initial H_2_ liberation to form an amido complex, which activates the alcohol *via* O–H proton transfer to generate an alkoxide intermediate. Subsequent β-hydride elimination releases the aldehyde, followed by formation of the hydrazone and base-induced Wolff–Kishner reduction to release N_2_ and furnish the deoxygenated product (Scheme 9).

**Scheme 9:**
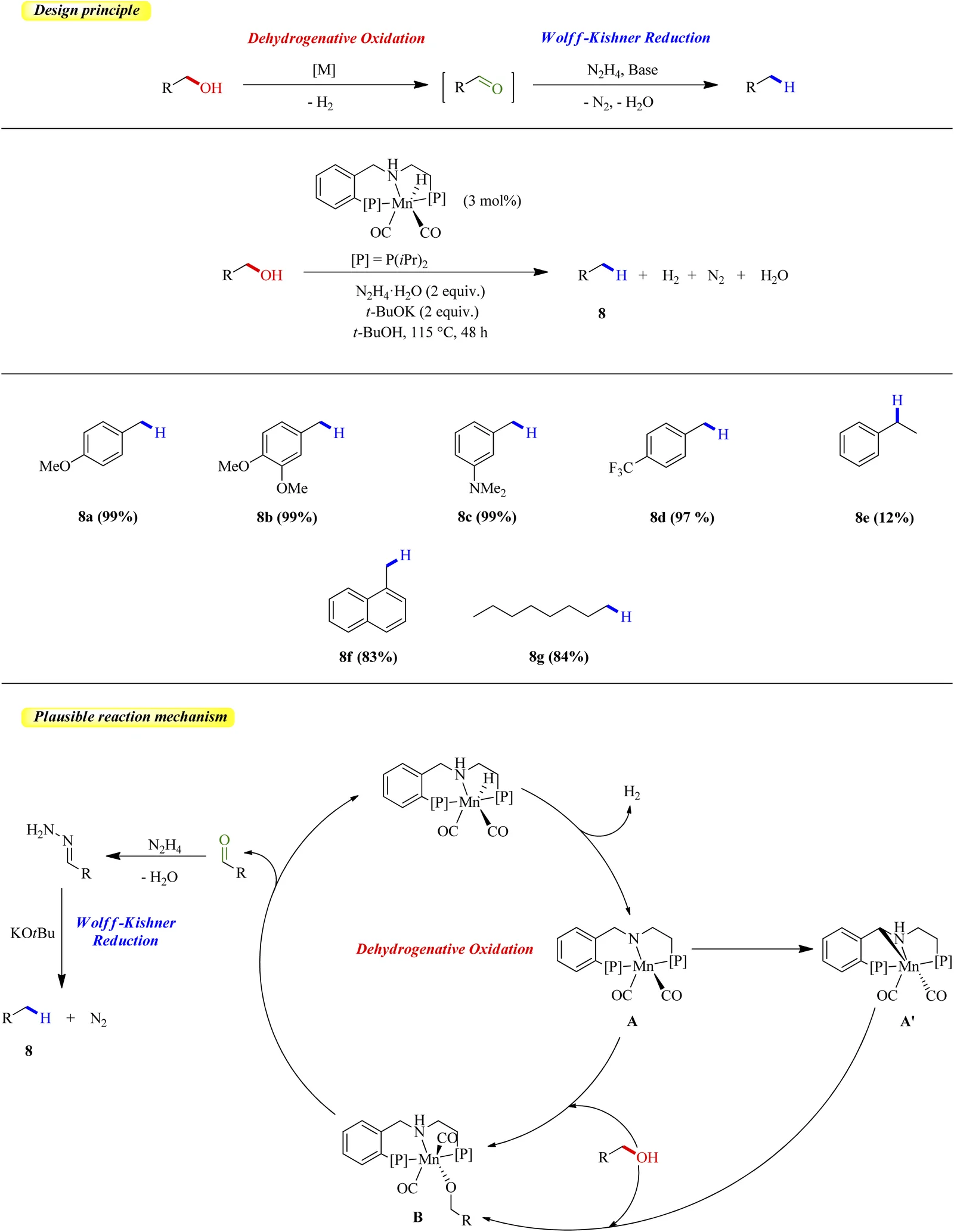
Manganese-catalysed direct deoxygenation of alcohols.

A safe and scalable continuous-flow Wolff–Kishner method was developed by Znidar *et al.*^49^ using the commercially available modular silicon carbide (SiC) reactor. This system allows for seamless transition capability all the way from laboratory-scale synthesis to full manufacturing scale. The importance of SiC was highlighted by a control experiment using a stainless-steel reactor coil, in which the presence of hydrazine immediately led to decomposition into nitrogen and water or ammonia at 150 °C, demonstrating that metal reactors are not compatible with these reaction conditions. Back-pressure regulator design was a crucial factor in controlling reaction performance because the excessive volume within the regulator caused N_2_ buildup and unpredictable pressure drops, while a regulator with minimal dead volume achieved complete conversion. This demonstrated that proper gas handling is as important as accurate temperature control for this process. Switching the solvent to lower-boiling methanol instead of high-boiling carbitol required heating up to 200 °C and 50 bar, achievable only through the pressure-resistance capabilities of sintered SiC. This change increased the purity of the final compound and facilitated extraction over chromatographic isolation, achieving a ten-fold increase in productivity compared with the previously reported^41^ SiC microreactor approach.

Steady-state experiments at increasing substrate concentrations (up to 1.6 M) showed that conversion remained high throughout, but reliable, controlled product collection could only be guaranteed up to 1.3 M, since higher concentrations generated too much N_2_ gas for stable outlet flow. In a separate 8 h long-run demonstration at 1.3 M, the process delivered the reduction of α-tetralone in 81% yield with a space-time yield of 152 g L^−1^ h^−1^. Substrate tolerance was explored across a variety of ketones and aldehydes, obtaining yields in the 70–90% range for isolated products, with aldehydes requiring a dual-feed system to prevent premature precipitation of hydrazones. The pharmaceutical significance of this continuous-flow platform was exemplified by the preparation of an important CDK9 inhibitor intermediate relevant to AZD4573, where the continuous route enabled the completion of the process in less than 20 min with a yield of 80% and purity greater than 99%, outperforming the 40% yield of the batch version (Scheme 10).

**Scheme 10:**
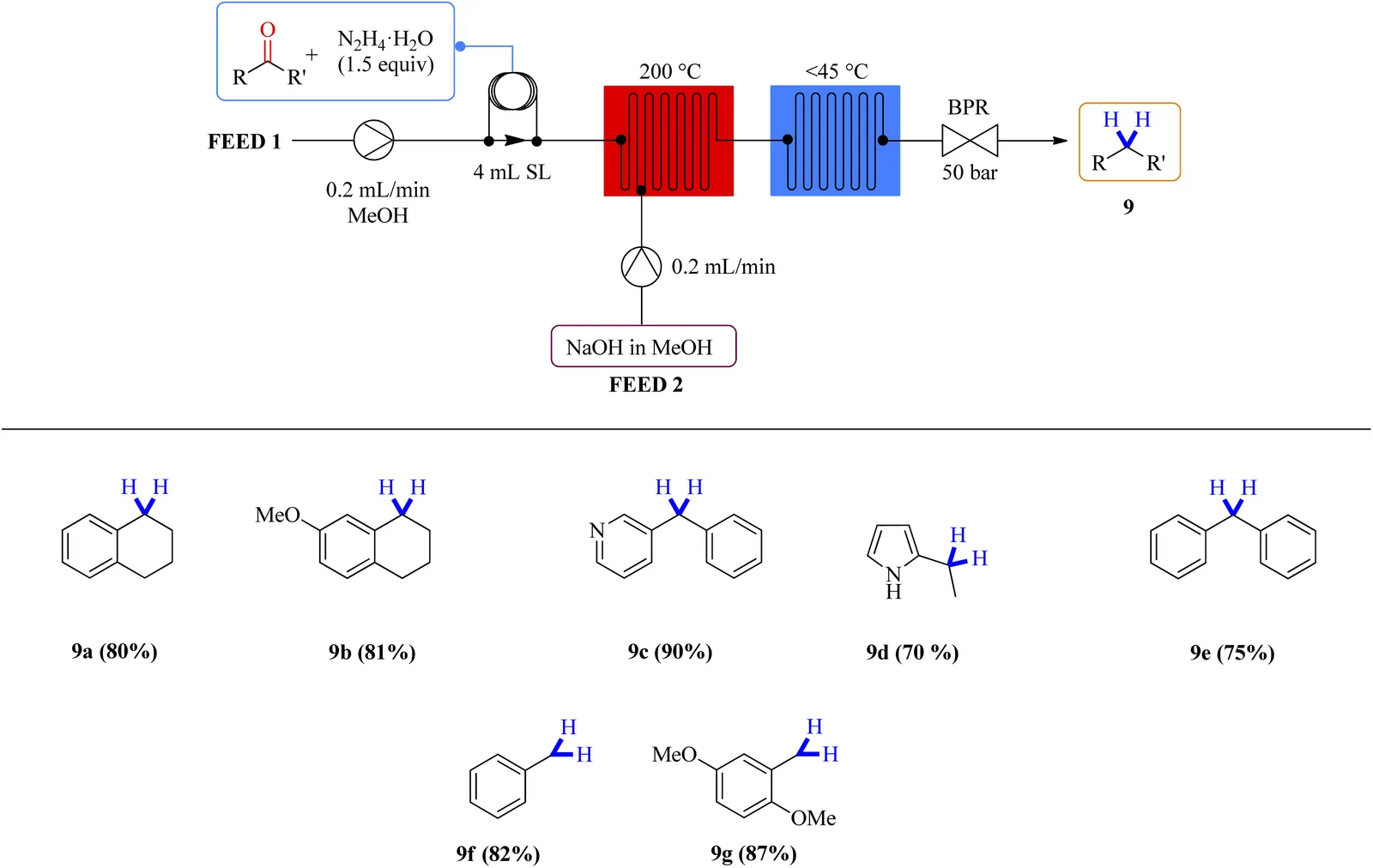
Continuous flow reduction of aldehydes and ketones using a two-feed silicon carbide reactor system.

Vasiljević *et al.*^50^ reported the first application of microwave-assisted Wolff–Kishner reduction to bile acid oxo derivatives, a substrate class previously unreported under these conditions. Applying the Huang-Minlon modification, the reduction of three bile acid oxo derivatives – 3,12-dioxo-5β-cholanic acid, 3,7,12-trioxo-5β-cholanic acid, and 3-oxo-5β-cholanic acid – was accomplished at 240 °C in 15 min under sealed-vessel microwave irradiation, compressing reaction times from hours to minutes relative to conventional heating. The combined high-temperature and high-pressure environment generated within the sealed vessel drove significantly higher conversions to 5β-cholanic acid, with yields of 92%, 67%, and 73% from the respective substrates.

The biological relevance of 5β-cholanic acid was further evaluated by testing its affinity for the glucocorticoid receptor ligand-binding domain using a yeast-based fluorescent assay, which revealed moderate binding affinity. Molecular docking predicted a binding geometry similar to known glucocorticoid receptor ligands, with conservation of key hydrogen-bonding interactions, though at a reduced binding energy relative to dexamethasone, consistent with the fewer hydrophilic groups present in the bile acid scaffold. The diminished affinity in comparison with prednisolone is not considered an anomaly, since docking analysis showed that dexamethasone – a closely related corticosteroid used as the co-crystallised reference ligand – forms additional hydrogen-bonding interactions in the glucocorticoid receptor binding site that 5β-cholanic acid, lacking the requisite hydrophilic groups, cannot replicate, a rationale likely extending to prednisolone given its structural similarity to dexamethasone. The importance of this finding is further substantiated by evidence demonstrating that 5β-cholanic acid rescues mitochondrial dysfunction in patient-derived cell models of Parkinson's disease, an effect shown to depend on glucocorticoid receptor activation in a neuronal model, thereby suggesting that even a modest receptor binding affinity might be adequate to activate the receptor activity in biological systems (Scheme 11).

**Scheme 11:**
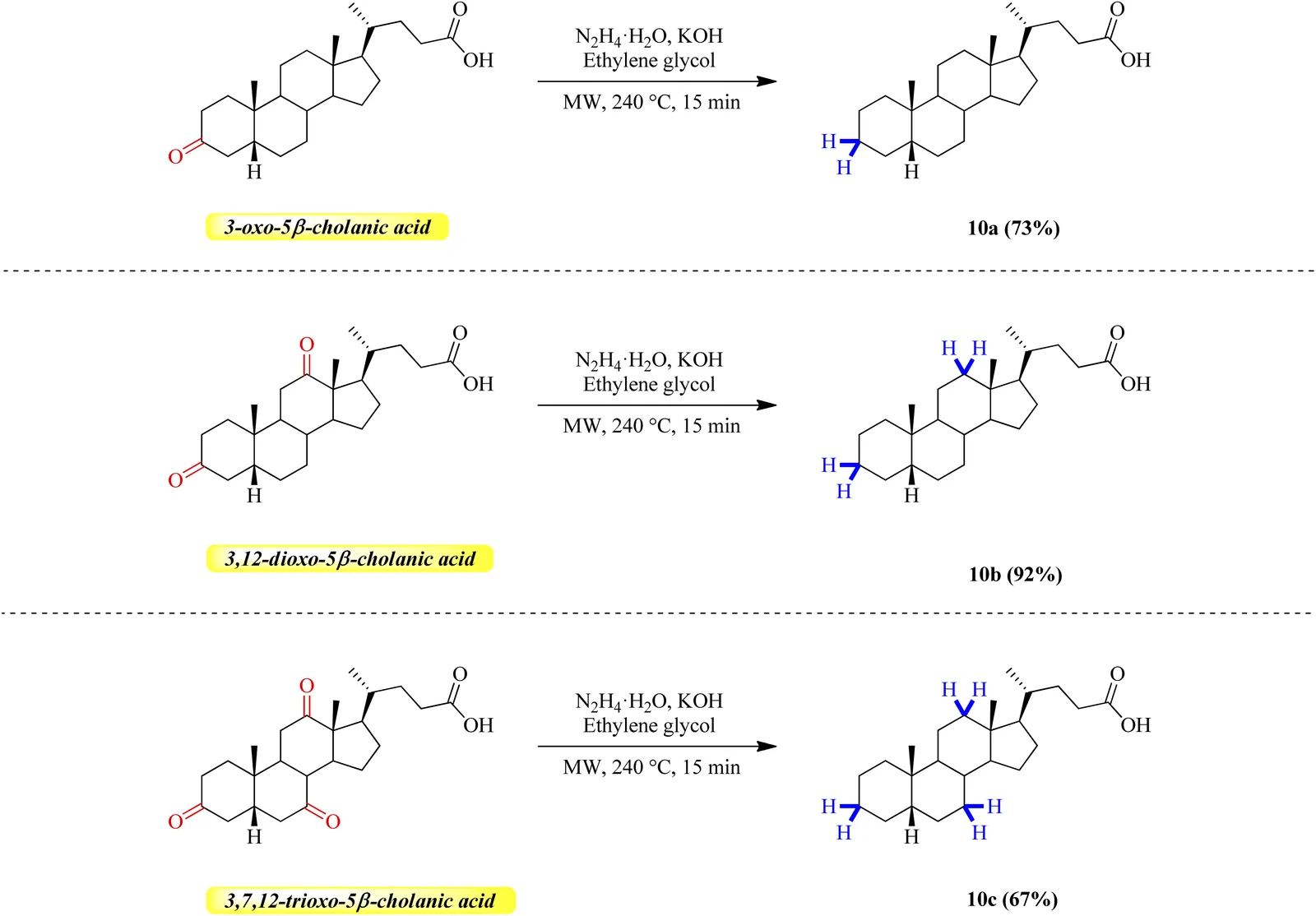
Microwave-assisted Wolff–Kishner reduction of bile acid oxo derivatives to 5β-cholanic acid.

According to Zhou and Chen,^51^ a Pd/C-catalysed, base-free reduction of aldehydes and ketones to produce hydrocarbons can be achieved using hydrazine hydrate as a reductant in an ethanol/water mixture at 100 °C. They note the importance of water in this process since the lack of it prevented any product formation. Hence, water plays an active role in the catalytic cycle instead of functioning merely as a co-solvent. Optimisation studies showed that, among palladium sources, Pd/C was highly compatible with hydrazine hydrate. The use of other palladium sources such as Pd(OAc)_2_ and PdCl_2_ led to the formation of palladium black and, therefore, loss of catalytic activity. A broad range of aldehydes and ketones served as substrates, providing excellent isolated yields reaching up to >99%. Cyclic ketones were found to resist reduction due to steric hindrance, since productive catalysis requires access to the carbonyl functionality.

Scale-up of the reaction to 10 g of benzophenone delivered diphenylmethane in 98.9% isolated yield, demonstrating the method's applicability beyond laboratory scale. The recovered Pd/C catalyst retained high catalytic efficiency across three cycles (98.9%, 96.7%, and 96.7% for cycles 1–3), showing only a marginal drop after the first cycle. The heterogeneous character of the catalyst thus ensures efficient and scalable use without any notable decrease in catalytic activity. The suggested reaction mechanism involves the formation of an active palladium hydride complex H–[Pd]–H in the presence of hydrazine hydrate; this complex reacts with the carbonyl substrate by nucleophilic addition to generate a palladium-bound complex. This palladium-bound complex then undergoes transformation to a second intermediate, from which elimination releases the deoxygenated hydrocarbon product, with regeneration of the active palladium catalyst completing the cycle. This pathway is mechanistically distinct from Wolff–Kishner chemistry in that it operates through metal-mediated hydride delivery rather than base-promoted hydrazone decomposition, thereby removing the requirement for strongly alkaline conditions (Scheme 12).

**Scheme 12:**
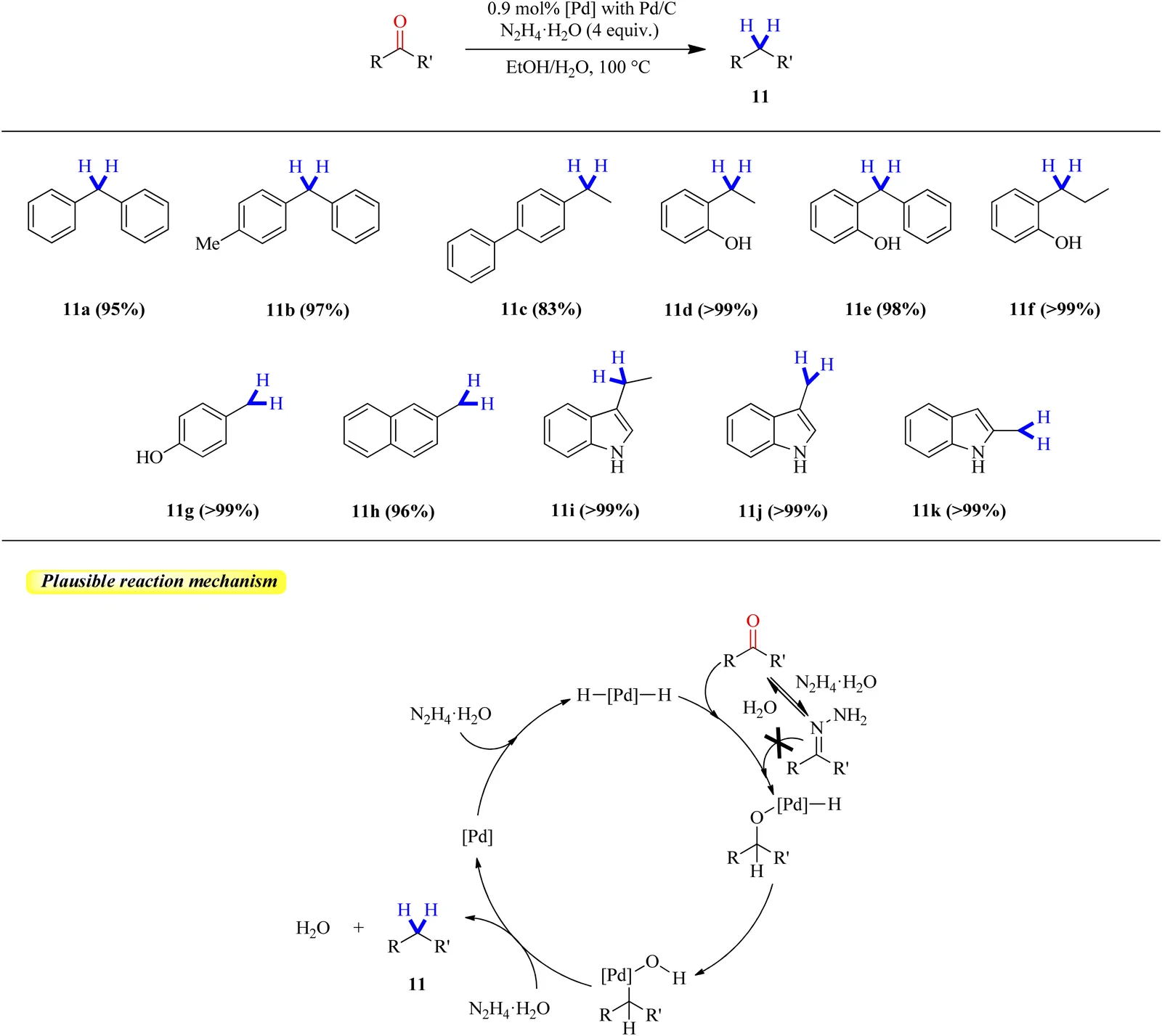
Base-free, recyclable Pd/C-catalysed reduction of ketones and aldehydes.

Chauhan *et al.*^52^ reported the first metal catalyst and hydrogen gas-free Wolff–Kishner reduction of 5-hydroxymethylfurfural (5-HMF) to 5-methylfurfuryl alcohol (MFA), a substrate class previously unexplored under these conditions. The inherent acidity of the 5-HMF (pH ∼3) was found to be responsible for the poor conversion, a limitation overcome by pre-neutralisation of the substrate to pH 7 prior to the reaction, which dramatically improved product yield. On a scale of 5 g, the procedure delivered alkyl furan products from various furfuraldehyde derivatives with isolated yields of 56–72% and product purity of more than 95%. This demonstrates that selectivity for aldehyde reduction is maintained despite structural variability in the furan skeleton, with no over-reduction of the functionalised furan ring. Additionally, the procedure was successfully demonstrated on a scale of 250 g using industrially valuable targets (12a and 12b), whereby stepwise addition of reagents proved crucial for controlling N_2_ release and maintaining consistent conversion rates. Yields of 69% and 70% were obtained respectively with product purity exceeding 90% without the need for further purification. This establishes that the protocol is directly scalable to a preparative level without sacrificing selectivity or yield.

The overall process consisted of a single-stage reaction, energy-saving *in situ* separation through distillation, reusability of the solvent, and generation of N_2_ and H_2_O as the only by-products. Altogether, these factors made the process low-waste, atom-economical, and superior compared to metal-catalysed hydrogenation. The 2-butanol solvent was entirely recovered, while the hydrazine hydrate decomposes into gaseous N_2_ and H_2_O, while the base was converted into readily marketable *t*-BuOH and KOH, meaning the process generated no significant hazardous waste streams (Scheme 13).

**Scheme 13:**
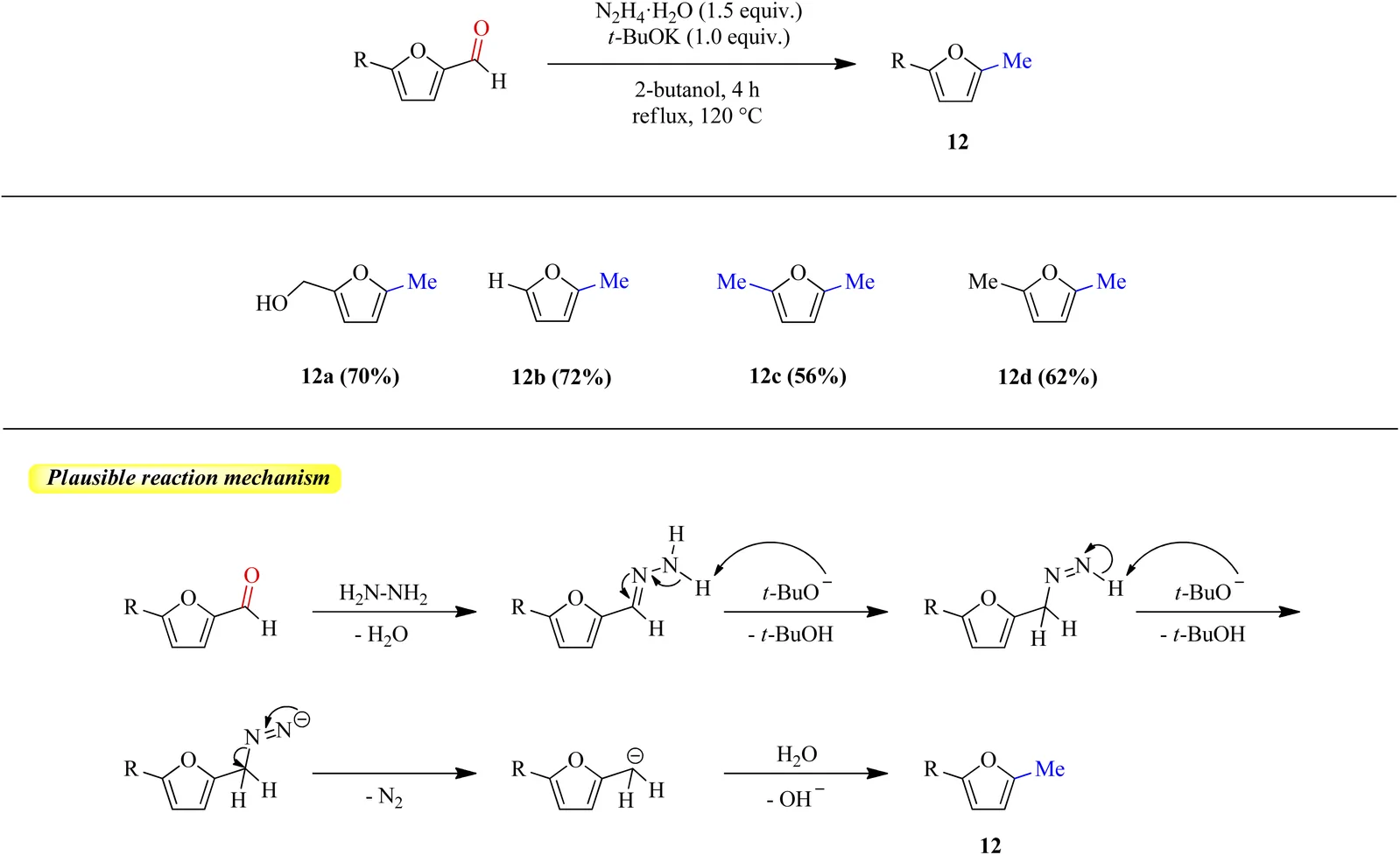
Metal catalyst and hydrogen gas-free selective reduction of substituted furfuraldehydes to alkyl furans.

Novichikhina and colleagues^53^ described a chemoselective Wolff–Kishner-type reduction of 4,4,6-trimethyl-4*H*-pyrrolo[3,2,1-*ij*]quinoline-1,2-diones to the corresponding 4*H*-pyrrolo[3,2,1-*ij*]quinolin-2(1*H*)-ones at the target position C-1, while leaving the other carbonyl group untouched. The reaction involved treating the diones with an aqueous solution of hydrazine hydrate under reflux at 115 °C for 1–3 h. The excess hydrazine was then distilled off under vacuum, giving the desired compounds in 67–82% yield. The reaction was carried out directly in aqueous hydrazine hydrate under reflux, avoiding the need for anhydrous conditions or additional organic solvents. A notable feature of this protocol is its chemoselectivity, as the reaction proceeded with preferential reduction at the C-1 carbonyl over the C-2 carbonyl. This selectivity was verified by the observation of characteristic methylene signals in the ^1^H NMR spectra and the absence of carbonyl signals in ^13^C NMR spectra. A notable limitation of this protocol involved the removal of excess hydrazine under reduced pressure, an operational step that presents scale-up challenges given the high volatility and reactivity of the hydrazine vapours. Nonetheless, the method remains a highly efficient and chemoselective gateway to the 2-one framework, opening pathways for further functional modification at the adjacent carbon position (Scheme 14).

**Scheme 14:**
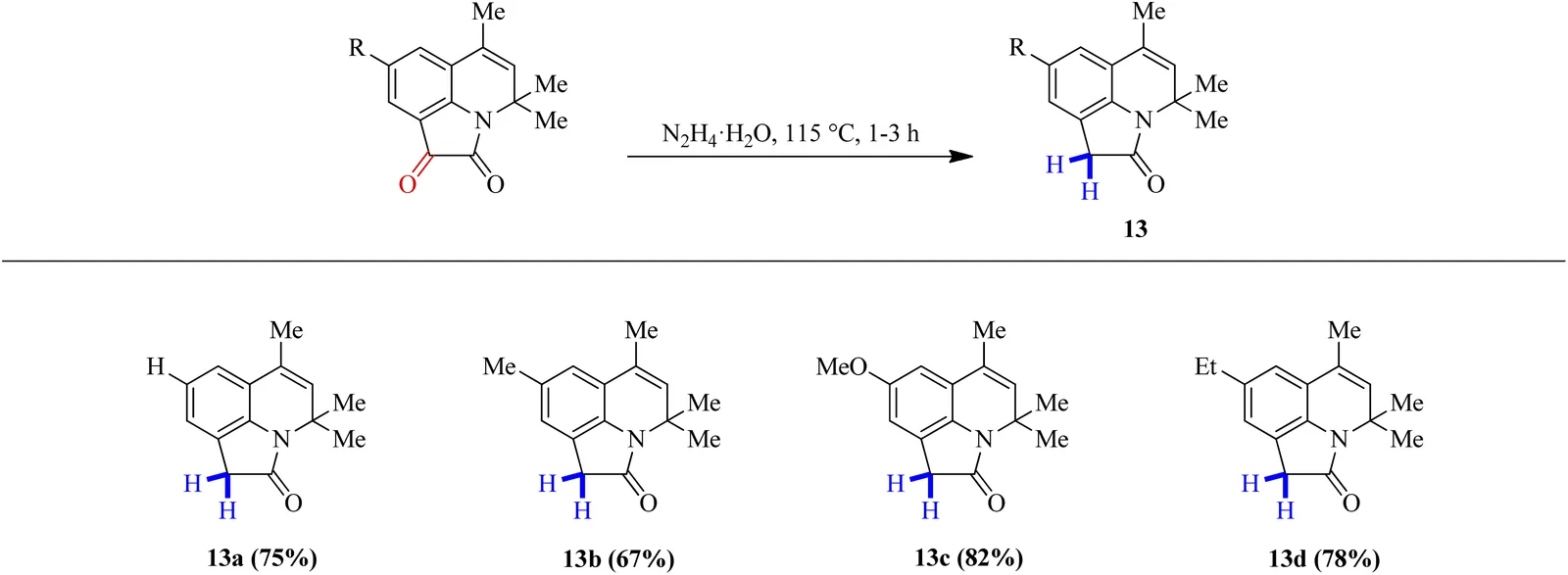
Chemoselective Wolff–Kishner reduction of pyrrolo[3,2,1-*ij*]quinoline-1,2-diones at C-1.

Gui and Li^54^ reported a ruthenium-catalysed Wolff–Kishner-type reduction of ketones under mild conditions using water or methanol as solvents. Catalyst optimisation revealed that the Ru-PNN complex, featuring a tridentate phosphine–nitrogen ligand, outperformed several ruthenium alternatives, including complexes bearing (di-*tert*-butylphosphino)ethylamine and bis(diisopropylphosphino)ethylamine ligands, as well as those featuring solely phosphine or nitrogen donor components. The PNN ligand was particularly effective because its electron-rich phosphine component facilitates denitrogenation of hydrazones, while the nitrogen component supports subsequent protonation, consistent with HSAB principles. Among solvents screened, methanol delivered the highest efficiency owing to the greater solubility of ketones in organic media, although alcohols in general could compete for ruthenium coordination sites and undergo dehydrogenation side reactions, an issue most pronounced with less optimal alcohol solvents. Water, meanwhile, remained the medium of choice for substrates better suited to aqueous conditions.

Studies showed that the mechanism of this reaction took place through a route similar to the classic Wolff–Kishner reduction. Here, ketones first reacted with hydrazine to form hydrazones, followed by metal-mediated denitrogenation reactions in basic media. The use of deuterium labelling showed that hydrazine monohydrate was the major proton donor, while protic solvents played a secondary role. Interestingly, the reaction proceeded efficiently even in the absence of an added base, suggesting that a ruthenium-hydride species facilitated the proton transfer process (Scheme 15).

**Scheme 15:**
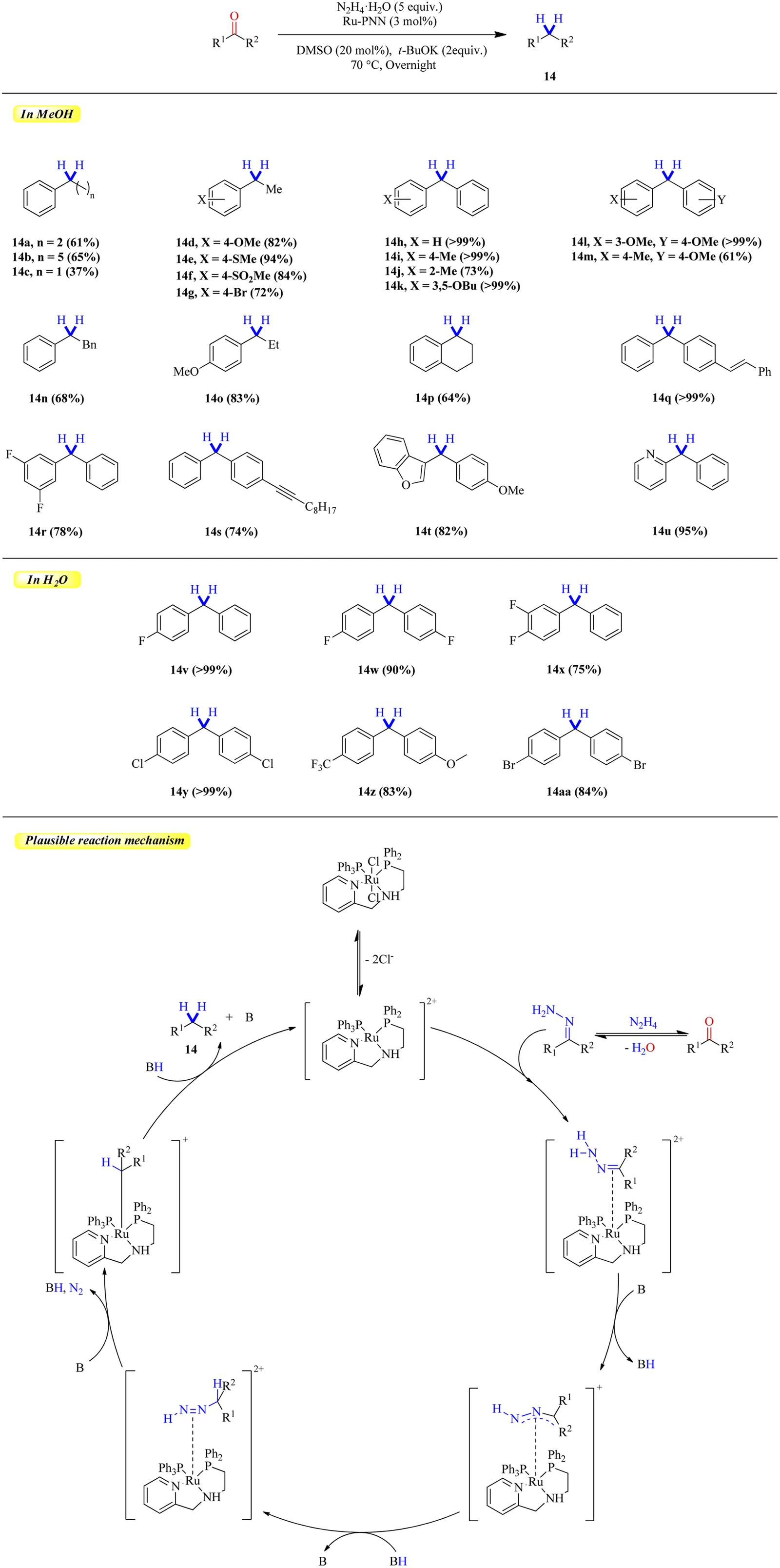
Ruthenium-catalysed deoxygenation of ketones.

Gui and Li^55^ further expanded their ruthenium-catalysed Wolff–Kishner-type platform, previously developed for ketone deoxygenation, toward a regiospecific deoxygenative deuteration strategy. Site-specific deuteration of this kind is of considerable interest to medicinal chemists, since replacing C–H bonds with stronger C–D bonds can slow metabolic breakdown through the kinetic isotope effect and thereby improve the pharmacokinetic profile of a drug. Conventional late-stage H/D exchange often struggles to provide such control because it gives mixtures of isotopologues and isotopomers, which is why methods that install deuterium regiospecifically are in demand. Building on their established Ru-PNN catalyst system, the authors reasoned that replacing regular hydrazine with deuterated hydrazine monohydrate (N_2_D_4_·D_2_O) could redirect the same mechanistic pathway to install deuterium selectively at the methylene position. Control experiments supported this reasoning, as reactions run in deuterated solvents without deuterated hydrazine gave only poor deuterium incorporation, showing that the hydrazine reagent itself, rather than the solvent, served as the primary deuterium source. Since protic solvents were thought to risk unwanted H/D back-exchange, aprotic solvents were screened instead, with THF and toluene both affording quantitative yields. Among various additives tested, CsF proved optimal, functioning not as a Lewis base catalyst but by sequestering trace water that would otherwise dilute deuterium incorporation. Deuterium content was quantified by crude ^2^D NMR analysis using DMSO-*d*_6_ as an internal standard, allowing direct assessment of isotopic incorporation without prior purification.

The reaction showed good applicability towards diaryl ketones, where the effect of electronic variation among substituents shows little impact on reactivity. This was reinforced by *ortho*-substituted substrates, where steric hindrance near the carbonyl group did not significantly reduce yield. Mechanistically, the ketone first condensed with deuterated hydrazine to form a deuterated hydrazone. Following deprotonation, the intermediate coordinated to ruthenium through two distinct binding modes, underwent a second deuteration, and then spontaneously denitrogenated to generate a carbanion that was subsequently deuterated, the hydrazine reagent being the only potential deuterium source. The aprotic medium thus prevented back-exchange, enabling high deuterium incorporation at the methylene position. The narrower substrate scope relative to the non-deuterated process may reflect a higher barrier for dedeuteration *versus* deprotonation (∼1.5 eV, as cited by the authors) or the lower solubility of ketones in the required aprotic solvents (Scheme 16).

**Scheme 16:**
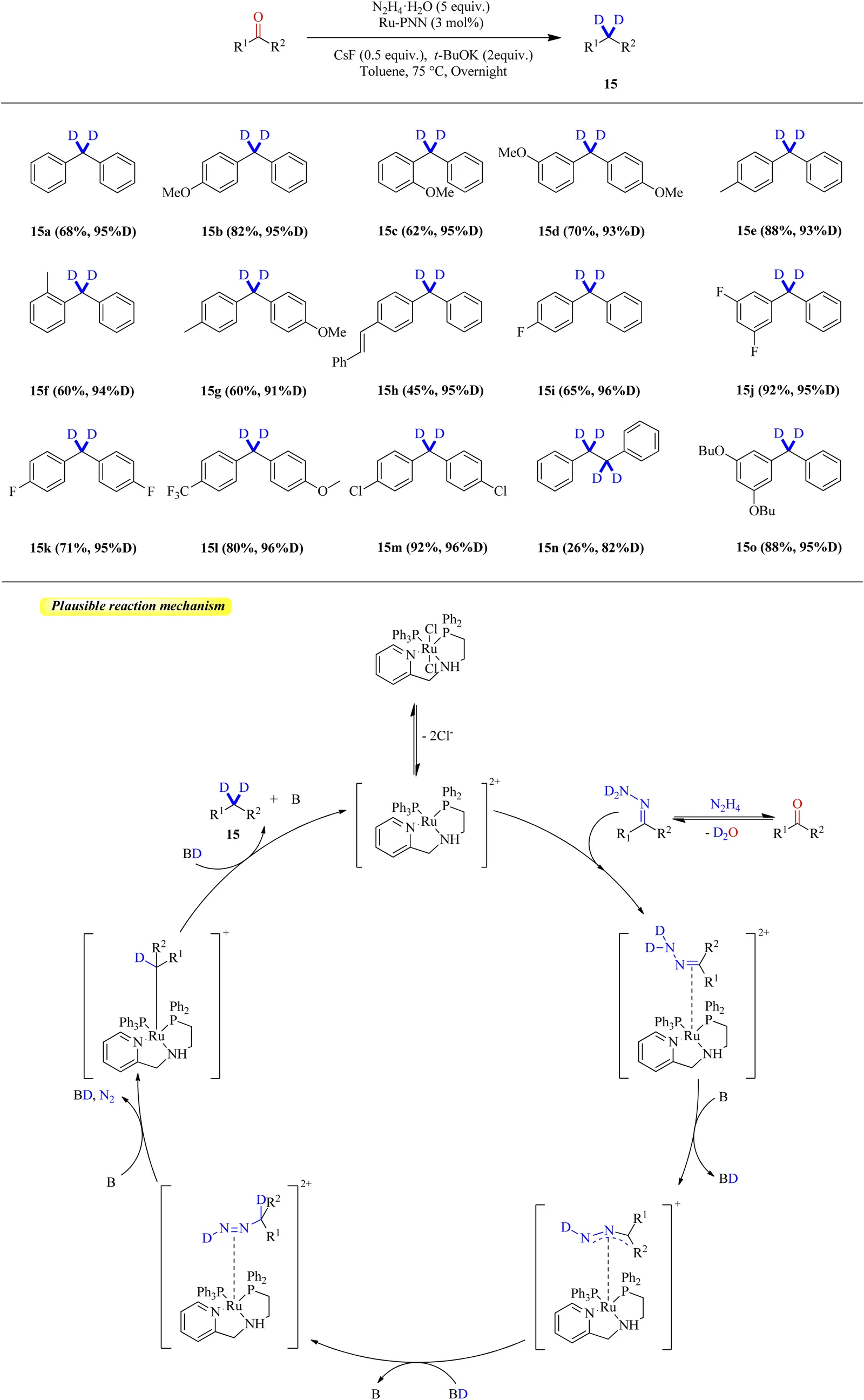
Ruthenium-catalysed deoxygenative deuteration of ketones to deuterated diarylmethanes.

Using the bench-stable alkynyl hydrazones as starting materials, Jamwal *et al.*^56^ successfully developed a method for synthesising allenoates *via* a DABCO-promoted Wolff–Kishner reduction followed by an isomerisation reaction. Firstly, the alkynyl hydrazone substrate reacted with the base 1,4-diazabicyclooctane (DABCO), giving an intermediate 3-alkynoate compound, which further participated in a 1,3-proton shift and produced the thermodynamic allenoate product in CH_3_CN at ambient temperature in yields up to 80%. The scope of substrates extended beyond simple aryl groups to pharmaceutical scaffolds. Allenoates from substrates such as ibuprofen (16k, 17k), ethinylestradiol (16l), and cholesterol (16m, 17m) were synthesised in moderate to good yields. In contrast to classical methods utilising unstable ketene compounds, diazo esters, or stoichiometric phosphines, here N_2_ gas was the only side product generated. It is worth noting that the allenoates were isolated along with the isomeric 3-alkynoates in ratios ranging from approximately 5 : 1 to 11 : 1, indicating substantial but incomplete chemoselectivity towards the allenoate product. Rather than the classical carbonyl-to-methylene conversion, the Wolff–Kishner reduction here acted as a stepping stone, where dinitrogen extrusion drove the formation of an alkynoate intermediate on its way to the final allenoate product (Scheme 17).

**Scheme 17:**
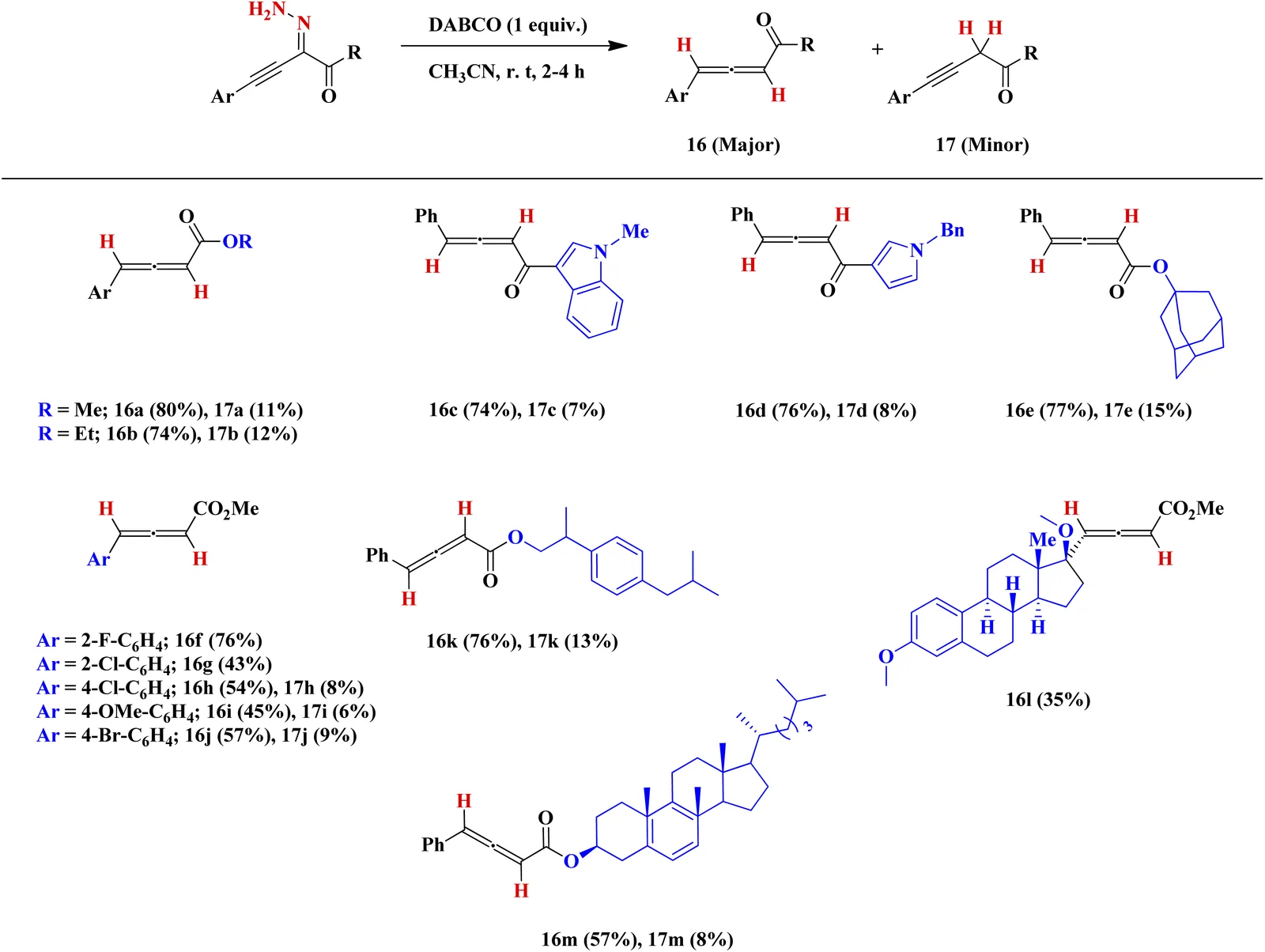
DABCO-promoted Wolff–Kishner reduction/isomerisation of alkynyl hydrazones to allenoates.

Chahal *et al.*^57^ described a metal-free and photocatalyst-free TBHP-based cross-dehydrogenative coupling reaction leading to highly valuable bioactive indeno-quinolinones using water as a solvent. These results addressed some shortcomings of methods suffering from the presence of intermediates, poor selectivities, and several-step processes involving pre-functionalisation of the starting compounds. The simple procedure, high substrate compatibility, and remarkable functional group tolerance made the developed strategy highly promising for future sustainable drug design based on direct preparation of selective 5-HT_3_ receptor ligands and TAS-103 derivatives undergoing clinical trials. Further applications of obtained indeno-quinolinone derivatives included their conversion into various bioactive structures. In particular, the 7*H*-indeno[2,1-*c*]quinolin-7-one could be successfully transformed to 7*H*-indeno[2,1-*c*]quinoline (18a) and 9-methoxy-7*H*-indeno[2,1-*c*]quinoline (18b) using 2-ethoxyethanol as solvent and hydrazine hydrate as a reducing agent at 100 °C for 4 hours with up to 92% yield. The key feature of the described process was the absence of the strongly basic environment typical for classical Wolff–Kishner methodology – most likely because the coordinating and protonic properties of the employed solvent allowed hydrazone formation and subsequent nitrogen expulsion under comparatively mild heating, preserving the integrity of the fused polycyclic framework (Scheme 18).

**Scheme 18:**
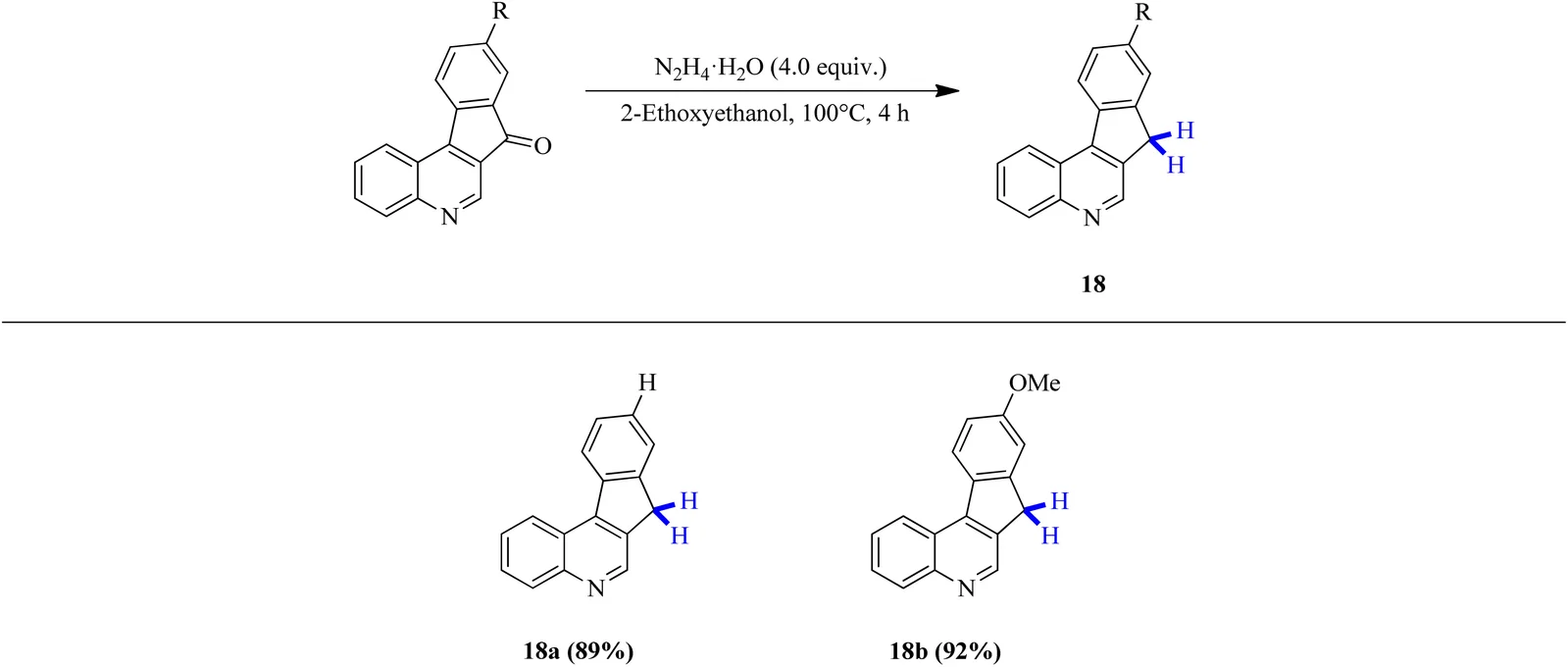
Wolff–Kishner reduction of indeno-quinolinones to indeno-quinolines in 2-ethoxyethanol.

## 5. Key limitations and potential solutions

Despite substantial progress in the development of gentler and more economical protocols for Wolff–Kishner reductions, substrate selectivity stands as a major challenge faced by almost all current versions of this process. Most current methods, such as those based on the use of ruthenium-, palladium-catalysis, or even flow processes, show strong preference for aromatic and diaryl ketones, whereas aliphatic ketones, especially bulky ones, give very poor yields or fail entirely. Halogenated substrates remain challenging due to competing dehalogenation side reactions, which have been observed under both heterogeneous palladium and continuous-flow conditions.^58–60^ This limitation severely narrows the method's utility for preparing pharmaceutically relevant scaffolds. To address this and truly expand the substrate scope, a viable strategy involves combining systematic ligand screening in transition-metal-catalysed methods with computational modelling to predict substrate-specific activation barriers.^61^

A second key issue concerns reagent and operational needs, which have yet to be fully addressed in modernisations of Wolff–Kishner reduction techniques. While most contemporary methods have successfully lowered hydrazine requirements to improve reagent efficiency, ruthenium, palladium, and solar-driven systems are notable exceptions that still require higher loadings. This is a critical drawback for techniques that are otherwise considered the most conceptually advanced departures from traditional Wolff–Kishner chemistry. One of the main disadvantages associated with transition metal-catalysed reactions is their dependence on complex ligands, including dmpe and PNN compounds, which necessitate multi-step synthesis processes. Continuous flow systems involve the need for an ongoing quenching process, as it is essential to eliminate the excess of hydrazine from the reaction mixture, introducing extra processing steps.^62^ Retaining the efficiency of state-of-the-art approaches while overcoming these drawbacks may be achievable through stable hydrazine alternatives, such as Myers' silylhydrazine, alongside the implementation of catalytic bases and inline continuous scavenging systems for flow chemistry.^63^

The issue of scalability also remains a significant practical challenge for the implementation of current state-of-the-art Wolff–Kishner chemistry from laboratory-scale studies towards preparative and industrial applications.^41^ The vast majority of substrate scope studies carried out in the surveyed literature utilise substrate amounts on a sub-millimolar scale and, upon scale-up, have encountered problems with yield loss and purity degradation. Regarding catalyst recycling, the number of recycling runs that has been reported, if at all, never exceeds three cycles and has only been validated using a model substrate rather than through broader substrate scope. Concerning flow systems, the productivity issue can be attributed to the uncontrolled release of dinitrogen gas at increased substrate concentrations, whereas the reactor capacity is still in the microliter volume range and there are no approaches available for numbering-up or parallelisation.^64,65^ Adoption of modular continuous flow platforms with integrated gas–liquid separation, real-time pressure management, and scalable silicon carbide reactor technology represents the most promising engineering solution to these throughput limitations.^66^

Although there are many examples of innovative developments in modern Wolff–Kishner reactions, the validation for their use in pharmaceutically relevant molecules is quite limited. Most investigations concerning substrate scope utilise relatively simple structures of aryl ketones and aldehydes; however, more complex structures of natural products, stereospecific scaffolds, and multi-functional drug-like molecules remain largely unexplored.^67^ In cases where pharmaceutical applicability is proven, such as in the synthesis of a precursor for a CDK9 inhibitor and the deoxygenation of bile acids, this has only been done by a few specific targets but not systematically assessed. Closer collaboration between synthetic methodology groups and medicinal chemistry, alongside deliberate validation against drug-like substrate libraries, would substantially accelerate the translation of modern Wolff–Kishner chemistry into applied pharmaceutical synthesis.^67,68^

## 6. Conclusions and future directions

Wolff–Kishner reduction has been quite a durable reaction, progressing through the development from a very harsh technique into a highly diversified one, where selectivity, sustainability, and scalability issues compete equally with reaction efficiency as key drivers for further progress. Not only has there been progressive optimisation, but there has also been a conceptual breakthrough that will increasingly guide researchers in designing deoxygenation using hydrazone-based chemistry by taking the mechanistic insight into account.^69^ It is important to note that the effectiveness of this century-old approach in its ability to adjust and fulfil the needs of modern synthesis is reflected in its timeless strategic importance in academia and industry alike.

The mechanistic understanding, provided by kinetic, isotopic, and computational investigations, has been extremely helpful for the development of modern protocols. The insight that the pathway is dictated by anionic charge stabilisation at the carbon terminal, and that the reaction can take place *via* either concerted or stepwise pathways based on the structure of the substrate, has enabled the design of catalysts that work in increasingly mild conditions. However, the exact process behind certain new catalytic variants that have been discovered still requires further investigation. Bridging this gap with thorough rigorous experimental and computational studies will be imperative.^70^ Overcoming the empirical and substrate-based approach to optimise and stabilise the catalysts is important for predictive catalyst design.^70,71^

Despite all of these achievements, there still remain numerous intertwined obstacles related to substrate scope, selectivity, and medicinal relevance which have yet to be solved. Specifically, aliphatic, sterically hindered, and halogenated substrates remain problematic across modern methodologies, while their drug-like relevance is yet to be demonstrated beyond isolated single-target demonstrations. Overcoming such shortcomings demands not only methodological breakthroughs but also conscious consideration of the structural complexity involved in medicinal chemistry, particularly structures that exhibit stereoselectivity and polyfunctional natural products where there are the greatest demands on chemoselectivity.^72–74^

Going forward, there are a number of directions worthy of investigation. The development of stable, non-toxic, and broadly reactive hydrazine surrogates, such as those devised by Myers using silylhydrazines, is a worthwhile endeavour, particularly as regards continuous-flow processing. Similarly, hydrazine-free protocols based on methyl hydrazinocarboxylate, which proceed through an isolable carbomethoxyhydrazone that is subsequently decomposed under basic conditions, offer a practical and safer alternative to neat hydrazine hydrate, a toxic, suspected carcinogenic and potentially teratogenic reagent, thereby avoiding the hazards associated with handling it in large quantities. Furthermore, the use of ruthenium-mediated deuteration *via* Gui and Li's method provides another means of harnessing the hydrazone motif, with potential for the production of labelled compounds of pharmaceutical interest.^75^ This is particularly attractive given the growing role of deuterium in medicine, from deutetrabenazine, the first deuterated drug approved by the FDA, to deucravacitinib, the first *de novo* deuterated drug to reach the market. Extending such hydrazone-based deoxygenative deuteration to pharmaceutically relevant carbonyl compounds could therefore meet the continuing need for site-specific labelling.^76,77^ The application of computational tools and machine learning algorithms for predicting mechanistic pathways based on substrates may contribute to the rapid creation of new methods in synthetic procedures.^78^ The Wolff–Kishner reduction is far from a settled topic. It remains a highly relevant tool in modern organic synthesis and studying how it works continues to open doors to new scientific breakthroughs.

## Author contributions

Charan D.C.: comprehensive literature survey, data curation, drafting of the manuscript, manuscript writing. Nitinkumar S. Shetty: review, editing, supervision, critical review of the manuscript, overall guidance.

## Conflicts of interest

There are no conflicts to declare.

## Data Availability

No primary research results, software or code have been included, and no new data were generated or analysed as part of this review. All data discussed are derived from previously published literature sources, which are appropriately cited throughout the manuscript.

## References

[cit1] Li J., Huang C. Y., Li C. J. (2022). Deoxygenative Functionalizations of Aldehydes, Ketones and Carboxylic Acids. Angew. Chem., Int. Ed..

[cit2] Cao J., Perlmutter P. (2013). “One-Pot” Reductive Lactone Alkylation Provides a Concise Asymmetric Synthesis of Chiral Isoprenoid Targets. Org. Lett..

[cit3] Magano J., Dunetz J. R. (2012). Large-Scale Carbonyl Reductions in the Pharmaceutical Industry. Org. Process Res. Dev..

[cit4] Li L., Chen Z., Zhang X., Jia Y. (2018). Divergent Strategy in Natural Product Total Synthesis. Chem. Rev..

[cit5] Schwob T., Kunnas P., de Jonge N., Papp C., Steinrück H.-P., Kempe R. (2019). General and Selective Deoxygenation by Hydrogen Using a Reusable Earth-Abundant Metal Catalyst. Sci. Adv..

[cit6] Palkovits R., Delidovich I. (2018). Efficient Utilization of Renewable Feedstocks: The Role of Catalysis and Process Design. Philos. Trans. R. Soc., A.

[cit7] Zhu R., Reddy R., Ding M., Xu M., Deng C., Tadayon S., Li H., Depew K., Lane B. (2021). Development and Scale-Up of a Continuous Manufacturing Process for a Hydrazine Condensation Reaction. Org. Process Res. Dev..

[cit8] Sánchez-Viesca F., Berros M., Gómez R. (2018). A Complete and Sustained Clemmensen Reduction Mechanism. Am. J. Chem..

[cit9] Dub P. A., Ikariya T. (2012). Catalytic Reductive Transformations of Carboxylic and Carbonic Acid Derivatives Using Molecular Hydrogen. ACS Catal..

[cit10] Volkov A., Gustafson K. P. J., Tai C. W., Verho O., Bäckvall J. E., Adolfsson H. (2015). Mild Deoxygenation of Aromatic Ketones and Aldehydes over Pd/C Using Polymethylhydrosiloxane as the Reducing Agent. Angew. Chem., Int. Ed..

[cit11] Colletto C. (2026). Reductive Transformations with Sodium Borohydride and Their Applications. Org. Process Res. Dev..

[cit12] Zlatković D., Radulović N. (2017). Study of Lithium Aluminium Hydride Reduction of 5-Acetyl-1,6-dimethyl-4-phenyl-3,4-dihydropyrimidin-2(1*H*)-one. Facta Univ., Ser.:Phys., Chem. Technol..

[cit13] Lewis D. E. , Modern Adaptations of the Wolff–Kishner Reduction, in The Wolff–Kishner Reduction and Related Reactions, Elsevier, 2019, pp. 133–167, 10.1016/b978-0-12-815727-5.00005-3

[cit14] Wolff L. (1912). Methode zum Ersatz des Sauerstoffatoms der Ketone und Aldehyde durch Wasserstoff. Justus Liebigs Ann. Chem..

[cit15] Suntsov V., Lewis D. E. (2015). The Catalytic Decomposition of Alkylidenehydrazines as a Method for the Preparation of Hydrocarbons (Abridged) (N. Kishner, Zh. Russ. Fiz.-Khim. O-va. 1911, 43, 582–595). Bull. Hist. Chem..

[cit16] Todd D. , The Wolff–Kishner Reduction, in Organic Reactions, ed. R. Adams, John Wiley & Sons, New York, 1948, vol. 4, pp. 378–422

[cit17] Szmant H. H. (1968). The Mechanism of the Wolff–Kishner Reduction, Elimination, and Isomerization Reactions. Angew. Chem., Int. Ed. Engl..

[cit18] Szmant H. H., Harmuth C. M. (1964). The Wolff–Kishner Reaction of Hydrazones. J. Am. Chem. Soc..

[cit19] Huang M. (1946). A Simple Modification of the Wolff–Kishner Reduction. J. Am. Chem. Soc..

[cit20] Barton D. H. R., Ives D. A. J., Thomas B. R. (1955). A Wolff–Kishner Reduction Procedure for Sterically Hindered Carbonyl Groups. J. Chem. Soc..

[cit21] Grundon M. F., Henbest H. B., Scott M. D. (1963). The Reactions of Hydrazones and Related Compounds with Strong Bases. Part I. A Modified Wolff–Kishner Procedure. J. Chem. Soc..

[cit22] Cram D. J., Sahyun M. R. V. (1962). Room Temperature Wolff–Kishner Reduction and Cope Elimination Reactions. J. Am. Chem. Soc..

[cit23] Caglioti L., Cainelli G., Selva A. (1962). Nuovo metodo per ottenere olefine da chetoni α,β-insaturi-α,β-sostituiti. Chim. Ind..

[cit24] Caglioti L., Magi M. (1962). A New Approach to Δ^16^-Androstenes. Tetrahedron Lett..

[cit25] Furrow M. E., Myers A. G. (2004). Practical Procedures for the Preparation of *N-tert*-Butyldimethylsilylhydrazones and Their Use in Modified Wolff–Kishner Reductions and in the Synthesis of Vinyl Halides and gem-Dihalides. J. Am. Chem. Soc..

[cit26] Kuethe J. T., Childers K. G., Peng Z., Journet M., Humphrey G. R., Vickery T., Bachert D., Lam T. T. (2009). A Practical, Kilogram-Scale Implementation of the Wolff–Kishner Reduction. Org. Process Res. Dev..

[cit27] Wang S., Cheng B. Y., Sršen M., König B. (2020). Umpolung Difunctionalization of Carbonyls *via* Visible-Light Photoredox Catalytic Radical-Carbanion Relay. J. Am. Chem. Soc..

[cit28] Moffett R. B., Hunter J. H. (1951). The Wolff–Kishner Reduction of 11-Keto Steroids. J. Am. Chem. Soc..

[cit29] Huang M. (1949). Reduction of Steroid Ketones and Other Carbonyl Compounds by Modified Wolff–Kishner Method. J. Am. Chem. Soc..

[cit30] Barton D. H. R. (1950). The Conformation of the Steroid Nucleus. Experientia.

[cit31] Honda T., Murae T., Tsuyuki T., Takahashi T. (1978). 13α*H*-Olean-18-ene Derivatives. Forced Wolff–Kishner Reduction Products of 19-Oxoolean-12-ene Derivatives. Bull. Chem. Soc. Jpn..

[cit32] Caglioti L. (1966). The Reduction of Tosylhydrazones and of Acyl Tosylhydrazides. Tetrahedron.

[cit33] Caglioti L., Magi M. (1963). The Reaction of Tosylhydrazones with Lithium Aluminium Hydride. Tetrahedron.

[cit34] Cacchi S., Caglioti L., Paolucci G. (1974). The Mechanism of the Reduction of Tosylhydrazones with B_2_H_6_ and with NaBH_4_ in Aprotic Solvents. Bull. Chem. Soc. Jpn..

[cit35] Caglioti L., Grasselli P. (1964). A New Method for the Reduction of Aldehydes and Ketones to CH_2_ under Mild Conditions. Chem. Ind..

[cit36] Szmant H. H., Harnsberger H. F., Butler T. J., Barie W. P. (1952). Kinetics of the Wolff–Kishner Reaction of Diaryl Ketone Hydrazones. J. Am. Chem. Soc..

[cit37] Szmant H. H., Román M. N. (1966). The Effect of Dimethyl Sulfoxide on the Rate of the Wolff–Kishner Reaction of Benzophenone Hydrazone. J. Am. Chem. Soc..

[cit38] Szmant H. H., Alciaturi C. E. (1977). Mechanistic Aspects of the Wolff–Kishner Reaction. 6. Comparison of the Hydrazones of Benzophenone, Fluorenone, Dibenzotropone, and Dibenzosuberone. J. Org. Chem..

[cit39] Taber D. F., Stachel S. J. (1992). On the Mechanism of the Wolff–Kishner Reduction. Tetrahedron Lett..

[cit40] Yamabe S., Zeng G., Guan W., Sakaki S. (2014). Substrate Dependent Reaction Channels of the Wolff–Kishner Reduction Reaction: A Theoretical Study. Beilstein J. Org. Chem..

[cit41] Newman S. G., Gu L., Lesniak C., Victor G., Meschke F., Abahmane L., Jensen K. F. (2014). Rapid Wolff–Kishner Reductions in a Silicon Carbide Microreactor. Green Chem..

[cit42] Agee B., Mullins G., Biernacki J., Swartling D. (2014). Wolff–Kishner Reduction Reactions Using a Solar Irradiation Heat Source and a Green Solvent System. Green Chem. Lett. Rev..

[cit43] Agee B. M., Mullins G., Swartling D. J. (2013). Friedel–Crafts Acylation Using Solar Irradiation. ACS Sustainable Chem. Eng..

[cit44] Cranwell P. B., Russell A. T., Smith C. D. (2016). Methyl Hydrazinocarboxylate as a Practical Alternative to Hydrazine in the Wolff–Kishner Reaction. Synlett.

[cit45] Cranwell P. B., Russell A. T. (2016). A Hydrazine-Free Wolff–Kishner Reaction Suitable for an Undergraduate Laboratory. J. Chem. Educ..

[cit46] Dai X. J., Li C. J. (2016). En Route to a Practical Primary Alcohol Deoxygenation. J. Am. Chem. Soc..

[cit47] Fischer F., Siegle A. F., Checinski M., Fischer C., Kral K., Thede R., Trapp O., Hapke M. (2016). Synthesis of Naphthylpyridines from Unsymmetrical Naphthylheptadiynes and the Configurational Stability of the Biaryl Axis. J. Org. Chem..

[cit48] Bauer J. O., Chakraborty S., Milstein D. (2017). Manganese-Catalyzed Direct Deoxygenation of Primary Alcohols. ACS Catal..

[cit49] Znidar D., O'Kearney-McMullan A., Munday R., Wiles C., Poechlauer P., Schmoelzer C., Dallinger D., Kappe C. O. (2019). Scalable Wolff–Kishner Reductions in Extreme Process Windows Using a Silicon Carbide Flow Reactor. Org. Process Res. Dev..

[cit50] Vasiljević B. R., Petri E. T., Bekić S. S., Ćelić A. S., Grbović L. M., Pavlović K. J. (2021). Microwave-Assisted Green Synthesis of Bile Acid Derivatives and Evaluation of Glucocorticoid Receptor Binding. RSC Med. Chem..

[cit51] Zhou X. Y., Chen X. (2020). Recyclable Pd/C Catalyzed One-Step Reduction of Carbonyls to Hydrocarbons under Simple Conditions without Extra Base. Tetrahedron Lett..

[cit52] Chauhan A. S., Kumar A., Das P. (2021). Metal Catalyst and Hydrogen Gas-Free Selective Reduction of Biomass-Derived Substituted Furfuraldehyde to Alkyl Furan as a Key Biofuel Additive. Org. Process Res. Dev..

[cit53] Novichikhina N. P., Ashrafova Z. E., Ledenyova I. V., Podoplelova N. A., Panteleev M. A., Shikhaliev K. S. (2022). Synthesis and Anticoagulant Activity of New Functionalized 4*H*-Pyrrolo[3,2,1-*ij*]quinolin-2-ones. Russ. J. Org. Chem..

[cit54] Gui R., Li C. J. (2022). Ruthenium(II)-Catalyzed Deoxygenation of Ketones. Chem. Commun..

[cit55] Gui R., Li C. J. (2023). Regiospecific Deoxygenative Deuteration of Ketones *via* HOME Chemistry. Org. Chem. Front..

[cit56] Jamwal P., Sharma A., Gurubrahamam R. (2023). Decomposition of Alkynyl Hydrazones: Synthesis of Allenoates, Dihaloallenoates, and Angularly Fused Tricyclic Azepines. Org. Lett..

[cit57] Chahal K., Potluri V. R., Tomy D., Vashishtha K., Reddy K. R. (2025). Synthesis of Indeno-Quinolones and Their Functionalization toward 5-HT_3_ Receptor. J. Org. Chem..

[cit58] Cellier P. P., Spindler J. F., Taillefer M., Cristau H. J. (2003). Pd/C-Catalyzed Room-Temperature Hydrodehalogenation of Aryl Halides with Hydrazine Hydrochloride. Tetrahedron Lett..

[cit59] Li F., Frett B., Li H. Y. (2014). Selective Reduction of Halogenated Nitroarenes with Hydrazine Hydrate in the Presence of Pd/C. Synlett.

[cit60] You T., Wang Z., Chen J., Xia Y. (2017). Transfer Hydro-dehalogenation of Organic Halides Catalyzed by Ruthenium(II) Complex. J. Org. Chem..

[cit61] Poree C., Schoenebeck F. (2017). A Holy Grail in Chemistry: Computational Catalyst Design: Feasible or Fiction?. Acc. Chem. Res..

[cit62] Elder D. P., Snodin D., Teasdale A. (2011). Control and Analysis of Hydrazine, Hydrazides and Hydrazones: Genotoxic Impurities in Active Pharmaceutical Ingredients (APIs) and Drug Products. J. Pharm. Biomed. Anal..

[cit63] Gutmann B., Cantillo D., Kappe C. O. (2015). Continuous-Flow Technology: A Tool for the Safe Manufacturing of Active Pharmaceutical Ingredients. Angew. Chem., Int. Ed..

[cit64] Mallia C. J., Baxendale I. R. (2016). The Use of Gases in Flow Synthesis. Org. Process Res. Dev..

[cit65] Dong Z., Wen Z., Zhao F., Kuhn S., Noël T. (2021). Scale-up of Micro- and Milli-reactors: An Overview of Strategies, Design Principles and Applications. Chem. Eng. Sci.:X.

[cit66] Plutschack M. B., Pieber B., Gilmore K., Seeberger P. H. (2017). The Hitchhiker's Guide to Flow Chemistry. Chem. Rev..

[cit67] Kutchukian P. S., Dropinski J. F., Dykstra K. D., Li B., DiRocco D. A., Streckfuss E. C., Campeau L.-C., Cernak T., Vachal P., Davies I. W., Krska S. W., Dreher S. D. (2016). Chemistry Informer Libraries: A Chemoinformatics Enabled Approach to Evaluate and Advance Synthetic Methods. Chem. Sci..

[cit68] Boström J., Brown D. G., Young R. J., Keserü G. M. (2018). Expanding the Medicinal Chemistry Synthetic Toolbox. Nat. Rev. Drug Discovery.

[cit69] Li C. J., Huang J., Dai X. J., Wang H., Chen N., Wei W., Zeng H., Tang J., Li C., Zhu D., Lv L. (2019). An Old Dog with New Tricks: Enjoin Wolff–Kishner Reduction for Alcohol Deoxygenation and C–C Bond Formations. Synlett.

[cit70] Jorner K., Brinck T., Norrby P.-O., Buttar D. (2021). Machine Learning Meets Mechanistic Modelling for Accurate Prediction of Experimental Activation Energies. Chem. Sci..

[cit71] Zahrt A. F., Henle J. J., Rose B. T., Wang Y., Darrow W. T., Denmark S. E. (2019). Prediction of Higher-Selectivity Catalysts by Computer-Driven Workflow and Machine Learning. Science.

[cit72] Shenvi R. A., O'Malley D. P., Baran P. S. (2009). Chemoselectivity: The Mother of Invention in Total Synthesis. Acc. Chem. Res..

[cit73] Guillemard L., Kaplaneris N., Ackermann L., Johansson M. J. (2021). Late-Stage C–H Functionalization Offers New Opportunities in Drug Discovery. Nat. Rev. Chem..

[cit74] Cernak T., Dykstra K. D., Tyagarajan S., Vachal P., Krska S. W. (2016). The Medicinal Chemist's Toolbox for Late Stage Functionalization of Drug-like Molecules. Chem. Soc. Rev..

[cit75] Atzrodt J., Derdau V., Kerr W. J., Reid M. (2018). Deuterium- and Tritium-Labelled Compounds: Applications in the Life Sciences. Angew. Chem., Int. Ed..

[cit76] Lele M., Manchare A., Parit S., Gholap A. D., Jadhav K., Hatvate N., Paudel K. R., Rojekar S. (2026). Unveiling the Power of Deuterium in Drug Discovery: A Comprehensive Overview. MedComm.

[cit77] Dabbs J. D., Taylor C. C., Holdren M. S., Brewster S. E., Quillin B. T., Meng A. Q., Dickie D. A., Pate B. H., Harman W. D. (2024). Designing Chemical Systems for Precision Deuteration of Medicinal Building Blocks. Nat. Commun..

[cit78] Coley C. W., Green W. H., Jensen K. F. (2018). Machine Learning in Computer-Aided Synthesis Planning. Acc. Chem. Res..

